# Aptamer-based drug delivery for targeted therapy of imatinib-resistant gastrointestinal stromal tumor

**DOI:** 10.7150/thno.115496

**Published:** 2025-08-11

**Authors:** Tao Pan, Ming Wang, Linxi Yang, Yihan Zheng, Yuanding Liu, Yu Xiao, Xudong Qiu, Yanying Shen, Mahan Dawuren, Zhiqiang Ren, Keying Liu, Yang Sun, Lin Tu, Hui Cao, Weihong Tan

**Affiliations:** 1 Department of Gastrointestinal Surgery, Institute of Molecular Medicine (IMM), Department of Pathology, Ren Ji Hospital, Shanghai Jiao Tong University School of Medicine, Shanghai 200127, China.; 2 Zhejiang Cancer Hospital, Hangzhou Institute of Medicine (HIM), Chinese Academy of Sciences, Hangzhou 310022, China.

**Keywords:** gastrointestinal stromal tumor, imatinib resistance, KIT, aptamer-drug conjugate, tumor-targeted therapy

## Abstract

**Rationale:** Gastrointestinal stromal tumors (GIST), the most common mesenchymal tumors of the gastrointestinal tract, are primarily driven by activating mutations in the KIT intracellular segment. The standard treatment with imatinib frequently results in acquired resistance due to secondary mutations. Besides mutations, KIT is also overexpressed in GIST. An aptamer that specifically binds to the extracellular segment of KIT (unaffected by these mutations) was promising in drug delivery and may overcome imatinib resistance.

**Methods:** The microtubule inhibitor VcMMAE (mc-vc-PAB-MMAE) was conjugated with an optimized KIT-targeting aptamer (KIT-d) to generate an aptamer-drug conjugate (ApDC) named KIT-d-MMAE. This ApDC was then evaluated for its binding specificity, internalization via endocytosis, and cytotoxicity towards KIT-positive GIST cells. The therapeutic efficacy of KIT-d-MMAE was evaluated through both *in vitro* and *in vivo* experiments.

**Results:** KIT-d-MMAE exhibited specific binding and efficient internalization into KIT-positive GIST cells, including imatinib-resistant lines, inducing targeted cytotoxic effects. In animal studies, KIT-d-MMAE significantly suppressed tumor growth in GIST-T1 subcutaneous and liver metastasis models. Notably, in imatinib-resistant GIST-430/654 and multi-TKI-resistant patient-derived xenograft (PDX) models, KIT-d-MMAE demonstrated superior antitumor efficacy compared to imatinib. Additionally, therapeutic effects were also observed in genetically engineered mouse models, indicating effective inhibition of spontaneous tumor formation and progression.

**Conclusion:** The aptamer-based drug delivery not only provides an innovative approach to overcome drug resistance but also simplifies treatment regimens, offering new therapeutic hope and marking a significant advancement in targeted therapy for GIST patients.

## Introduction

Gastrointestinal stromal tumors (GIST) represent the predominant form of mesenchymal tumor. Globally, GIST incidence varies between 10 and 15 cases per million annually, which is significantly higher in East Asia compared to North America 1, 2. The prevailing view is that in most cases (approximately 75%-80%), GIST pathogenesis is attributed to activating mutations in KIT or PDGFRA, genes encoding transmembrane tyrosine kinase receptors. These mutations result in continuous ligand-independent signaling, which activates the Ras/Raf/MAPK, PI3K/AKT, and JAK-STAT3 pathways, ultimately leading to uncontrolled and aberrant tumor cell proliferation 3-5. Imatinib, a small molecule tyrosine kinase inhibitor (TKI), has been successful in treating GIST and has emerged as a model for targeted therapy, heralding a new era 6. Presently, imatinib is the recommended first-line treatment for most primary, as well as unresectable, metastatic, or recurrent advanced GIST patients 7. While imatinib marks a significant advance in GIST treatment, it has notable limitations. Its efficacy is significantly tied to specific mutation types; patients with exon 13 mutations in KIT, wild-type GIST, or exon 18 D842V mutation in PDGFRA show inherent resistance to imatinib 8. Additionally, about 50% of patients experience imatinib resistance within two years, causing recurrence or progression of GIST. This resistance often correlates with secondary intra-allelic mutations in KIT or PDGFRA 9, 10. Furthermore, TKIs lack cytotoxic effects and can't eliminate all GIST cells. Some GIST cells enter a dormant state, evading the effects of imatinib 11, 12. When drug treatment is discontinued or secondary tumor mutations occur, recurrence becomes likely 13, 14. To overcome imatinib resistance, newer TKIs like sunitinib, regorafenib, and ripretinib have been approved for sequential second to fourth-line GIST treatments 15-17. Resistance to all targeted treatments is inevitable, leading to mortality in many patients. Like ripretinib, the median progression-free and overall survival rates stand at only 6.3 and 15.1 months, respectively 17. These findings highlight the need to explore new targeted treatment approaches for advanced GIST.

Antibody-drug conjugates (ADCs) are a promising strategy for enhancing tumor-specific therapeutic targeting 18. Existing research on ADCs for GIST treatment has yielded encouraging preclinical outcomes 19, 20. Nonetheless, clinical results have fallen short of expectations, possibly attributed to the substantial molecular weight of antibodies, limiting their ability to deeply infiltrate tumor tissues 21, 22. Aptamers, short single-stranded oligonucleotides derived via SELEX (Systematic Evolution of Ligands by Exponential enrichment) from RNA or DNA libraries, exhibit high specificity and affinity similar to antibodies 23, 24. Their distinct advantages include minimal toxicity and immunogenicity, flexible structural modification, and a wide range of targets 25. These characteristics have propelled aptamers to the forefront of targeted drug delivery systems as classical recognition ligands 26. Aptamers can be conjugated to cytotoxic drugs, forming Aptamer-drug conjugates (ApDCs), which enhance the therapeutic efficacy and diminish the side effects compared to the administration of free drugs 27. Within targeted cancer therapy, both ApDCs and ADCs are utilized. Yet, ApDCs stand out by not aggregating and demonstrating enhanced stability, lower toxicity, and greater specificity 28, 29. Furthermore, they are simpler and more cost-effective to produce, offering significant advantages over ADCs in therapeutic applications 30, 31. To date, research on the application of ApDCs in GIST treatment remains unexplored.

**Scheme 1:**
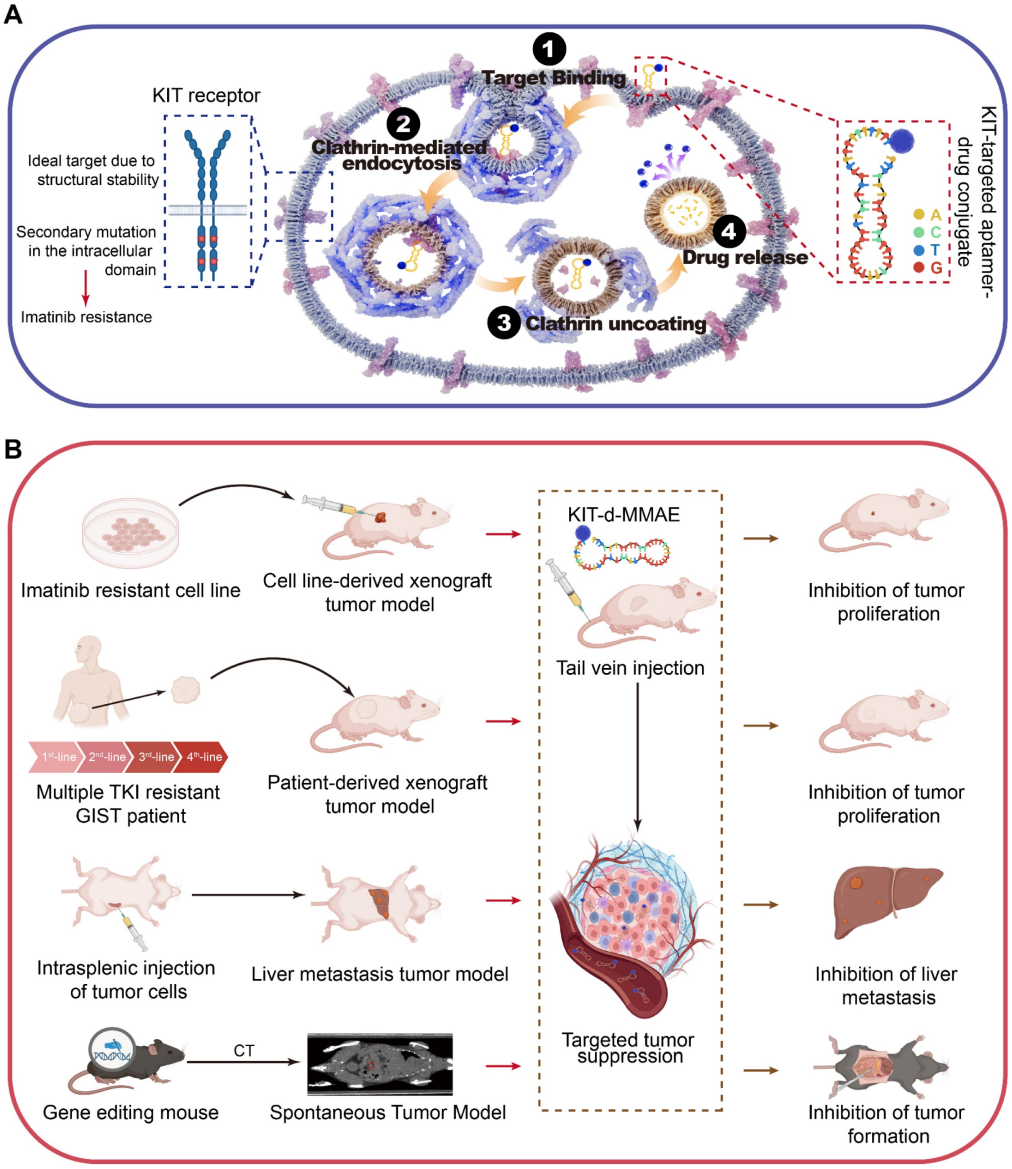
** Schematic diagram and application of KIT-d-MMAE.** (**A**) Schematic diagram showing the mechanism of KIT-d-MMAE uptake and release. (**B**) Application and anti-tumor effect of KIT-d-MMAE in four different tumor models.

Given the overexpression of KIT in GIST, its plasma membrane localization, and its secondary mutations are mainly located in the intracellular domain 32, we expected KIT to be an attractive target for drug delivery. KIT targeting aptamer was reported previously and targeted toxicity was observed in AML. In this study, we optimized and truncated the nucleotide aptamer targeting KIT and constructed an ApDC by conjugating it with VcMMAE. Results reveal that ApDC targets GIST by specific binding with KIT and subsequently internalizes into tumor cells to release the drug (Scheme 1A). Upon release, MMAE exerts significant cytotoxic effects, effectively inducing cell apoptosis thus inhibiting the proliferation of GIST cells. Moreover, the anti-tumor efficacy and biosafety of KIT-d-MMAE were observed in several tumor models (cell line-derived xenograft, patient-derived xenograft, liver metastasis tumor model, and spontaneous tumor model) (Scheme 1B). Thus, this study constructs an ApDC targeting KIT, which represents a promising new targeted therapeutic option for advanced and recurrent GIST.

## Experimental Section

### Synthesis of ApDC

KIT-d-MMAE was synthesized by coupling thiol-labeled KIT-d with maleimide-modified MMAE. Briefly, the thiol-labeled KIT-d was dissolved in DPBS buffer with 1 mM TCEP for 1 h. After the removal of TCEP, maleimide-modified MMAE with 5-fold equivalent in acetonitrile was added. The mixture underwent continuous stirring at ambient temperature for an overnight duration. Subsequently, it was processed through high-performance liquid chromatography (HPLC) for the purification of ApDCs.

### Animal experiments

All animals were maintained in a pathogen-free environment at the Renji Hospital (Shanghai, China) animal facility with controlled temperature, humidity, and light-dark cycle. All animal studies were reviewed and approved by the Ethics Committee of Renji Hospital (2018-029, RJ-2023-114A) and were performed by NIH guidelines.

For antitumor activities of ApDC in GIST cell line xenograft models, GIST-T1 cells (1*10^6^) or GIST430/654 (3*10^6^) were formulated as a 1:1 mixture with Matrigel (BD Biosciences) and were subcutaneously injected into BALB/c nude mice. The resulting tumors were measured twice a week and the total volume was using the following formula: (length × width^2^)/2, where length was the longest axis and width was the distance perpendicular to the length. For the GIST-T1 xenograft model, when the tumor volume reached 80-120 mm^3^, mice were randomized into six groups (n = 5 per group) and injected via tail vein with DPBS, KIT-d, SMCC-DM1, KIT-d-DM1, VcMMAE or KIT-d-MMAE (equivalent KIT-d-MMAE concentration = 4 mg kg^-1^) twice a week for 5 times. After treatment, all mice were killed and their tumor and major organs were collected and subjected to immunohistochemical and histological examinations. For GIST-430/654 xenograft model, when the tumor volume reached 80-120 mm^3^, mice were randomized into five groups (n = 5 per group) and administered DPBS, KIT-d, VcMMAE or KIT-d-MMAE (equivalent KIT-d-MMAE concentration = 4 mg/kg) via tail vein injection twice a week for 6 times or imatinib (50 mg/kg) orally once a day for 3 weeks. After treatment, all mice were killed and their tumor and major organs were collected and subjected to immunohistochemical and histological examinations.

For antitumor activities of ApDC in a GIST PDX model, GIST tissues from patients with multi-TKI resistance were implanted into nude mice. The resulting tumors were measured twice a week. When the tumor volume reached 80-120 mm^3^, mice were randomized into five groups (n = 5 per group) and administered DPBS, KIT-d, VcMMAE or KIT-d-MMAE (equivalent KIT-d-MMAE concentration = 4 mg/kg) via tail vein injection twice a week for 6 times or imatinib (50 mg/kg) orally once a day for 17 days. After treatment, all mice were killed and their tumor and major organs were collected and subjected to immunohistochemical and histological examinations.

For Antitumor activities of ApDC in a GIST spontaneous tumorigenesis model, female *Kit*^V558del/+^ mice 33 (4-6 months old; weight 25-30 g) were randomized into two groups (n = 5 per group) and administered DPBS or KIT-d-MMAE (4 mg/kg) via tail vein injection twice a week for 7 times. After treatment, all mice were killed and their tumor and major organs were collected and subjected to immunohistochemical and histological examinations.

For antitumor activities of ApDC in a GIST liver metastasis model, GIST-T1 cells (2*10^6^) were injected into the spleen to construct the liver metastasis model. Two weeks after tumor inoculation, mice were randomized into two groups (n = 7 per group) and administered DPBS or KIT-d-MMAE (4 mg/kg) via tail vein injection twice a week for 6 times. After treatment, all mice were kept for about 3 weeks and finally killed and their livers and other major organs were collected and subjected to histological examinations.

### Statistical analysis

All data are presented as the mean ± SD. GraphPad Prism 9.0 (GraphPad Inc., La Jolla, CA, USA) was used to display differences between two groups (Student's t-test) or among three or more groups (one-way ANOVA analysis followed by Fisher's LSD multiple comparisons). A p-value of < 0.05 was considered statistically significant. ns, not significant; ns = not significant. *P < 0.05, **P < 0.01, ***P < 0.001, ****P < 0.0001.

## Results

### Confirmation of KIT as a target for GIST

**Figure 1:**
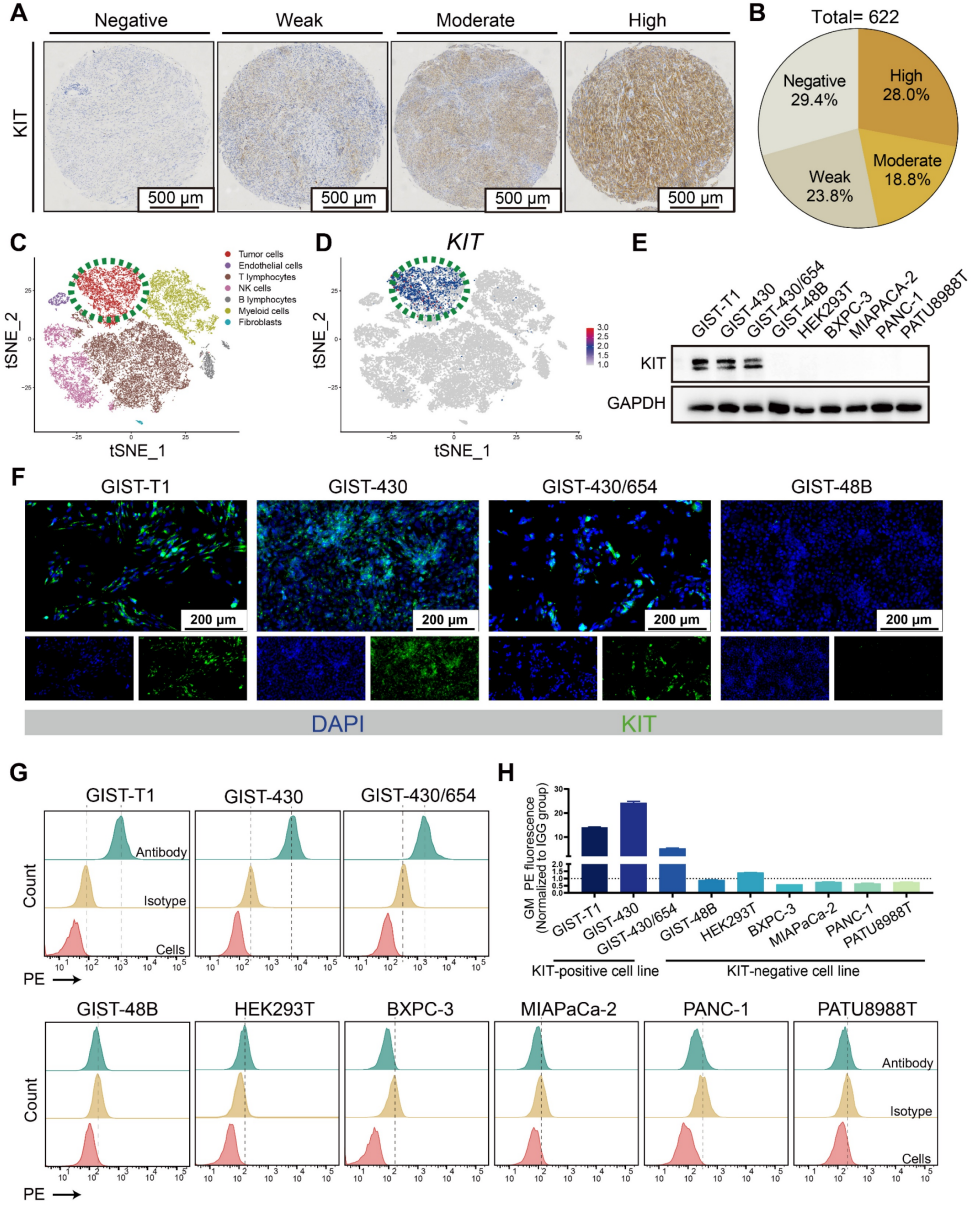
** KIT is a biomarker in GIST.** (**A**) Immunohistochemical stain of KIT in GIST tissues with specific antibody. KIT expression levels are categorized as negative, weak, moderate, and high. (**B**) KIT expression level was summarized according to categorization in Figure 1A (n = 622). (**C**) Unsupervised clustering of viable cells from two human GIST resections represented as a t-SNE plot. Each dot represents a single cell colored by cell type. (**D**) The t-SNE plot shows the expression of *KIT* in the GIST cells. The legend shows a color gradient of normalized expression. (**E**) The relative expression of KIT protein in GIST cell lines and other cell lines was studied with western blot. (**F**) The location of KIT in GIST cell lines was visualized with immunofluorescence. (**G**) The protein level of KIT on the cell surface was analyzed with flow cytometry. (**H**) Statistical analysis of the geometric mean fluorescence intensity in Figure 1G.

Mutation of KIT is reported in GIST patients, while whether it can function as a target for drug delivery needs to be further clarified. The tissue microarray comprised samples from 622 patients (Figure 1A-B). About 70% KIT positivity rate was detected, which aligns with previous literature reports 34, 35. In a reanalysis of a single-cell dataset of GIST 36, it was observed that KIT expression is predominantly found in the tumor cells (Figure 1C-D). Thereafter, we determined the KIT protein levels in various GIST cell lines (GIST-T1, GIST-430, GIST-430/654, and GIST-48B), HEK-293T, and various pancreatic cancer cell lines (BXPC-3, MIAPACA-2, PANC-1, and PATU8988T). Results showed that KIT was overexpressed in GIST-T1, GIST-430, and GIST-430/654, but showed negative expression in other cells (Figure 1E). Furthermore, immunofluorescence analysis exhibited strong KIT staining in GIST-T1, GIST-430, and GIST-430/654 cells (Figure 1F). Considering previous studies that identified KIT expression in GIST not exclusively on the cell membrane 37, we employed flow cytometry to assess the membrane level of KIT. A significant signal shift was observed in GIST-T1, GIST-430, and GIST-430/654 in antibody-treated groups compared to the isotype controls, while no notable shift was observed in other cells (Figure 1G-H). Together, these results demonstrated that KIT is a biomarker for GIST.

### Sequence optimization and binding ability test of KIT targeting aptamer

**Figure 2:**
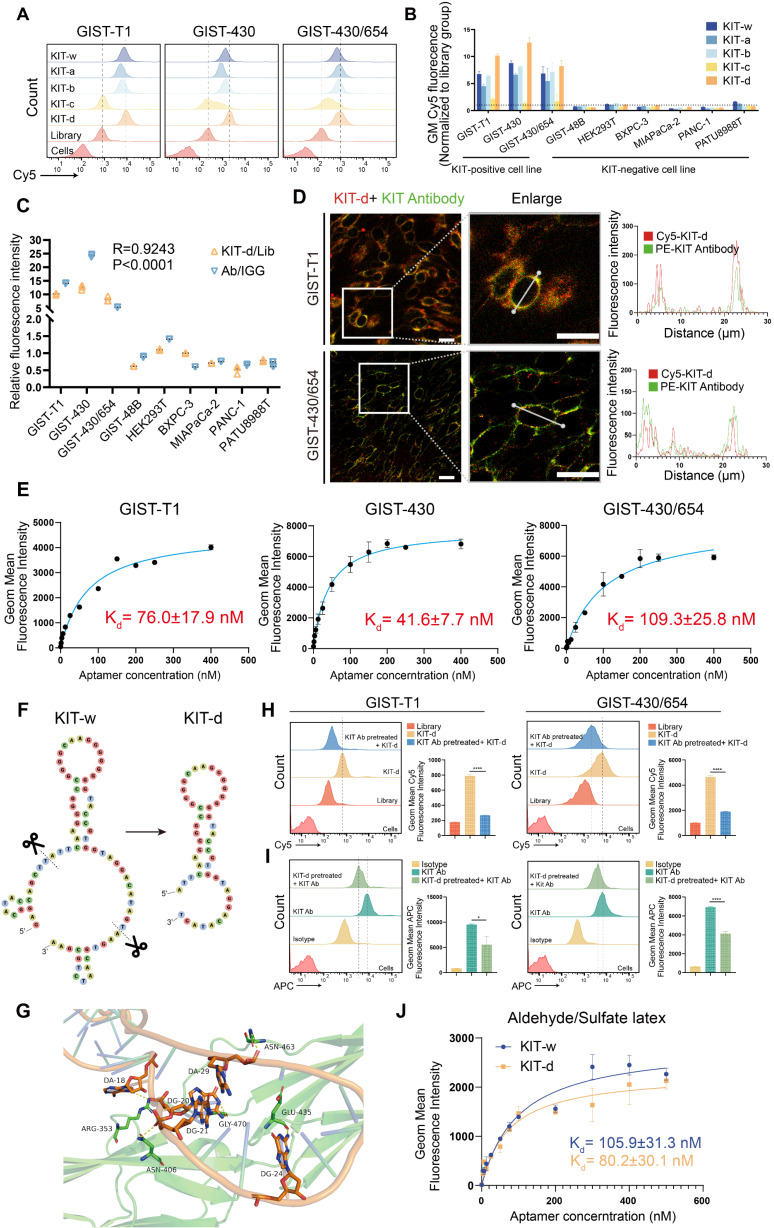
** Sequence optimization and binding ability test of KIT targeting aptamer. (A**) The binding ability of KIT targeting aptamer (Cy5 labeled) to GIST-T1, GIST-430 and GIST 430/654 cells. (**B**) Statistical analysis of the geometric mean fluorescence intensity in Figure 2A and Figure S1. (**C**) The relative fluorescence intensity and the correlation between KIT-d and anti-KIT antibody were analyzed with flow cytometry. (**D**) Micrographs showing the binding of Cy5-labeled KIT-d (red) and PE-labeled KIT antibody (green) to GIST-T1 and GIST-430/654 cells, and the fluorescence intensity in single cells was quantified. Scale bar = 20 μm (**E**) The binding affinities of KIT-d to GIST-T1, GIST-430, and GIST-430/654 cells have been analyzed, and the apparent Kd constants have been determined. (**F**) Secondary structures of KIT-w and KIT-d were predicted by RNAfold. (**G**) Close-up view of the predicted binding site between KIT-d and KIT protein. (**H-I**) KIT targeting antibody and KIT-d aptamer competitively bind to KIT protein on GIST-T1 and GIST-430/654 cells. (**H**) Binding ability of KIT-d to GIST-T1 and GIST-430/654 cells with or without KIT antibody pretreatment. (**I**) Binding ability of KIT antibody to GIST-T1 and GIST-430/654 cells with or without KIT-d pretreatment. (**J**) The binding affinity of KIT-d to KIT protein was assessed using flow cytometry, with the protein coated on aldehyde/sulfate latex beads.

A 77nt DNA aptamer target KIT (termed as KIT-w in our study) was reported previously 38. Flow cytometry was performed to verify its binding ability to KIT, results indicated that KIT-w exhibited strong affinity towards KIT-positive cell lines, and it demonstrated lower affinity to KIT-negative cell lines (Figure 2A-B and Figure S1). To minimize synthesis costs and enhance efficiency we designed four truncated variants: KIT-a, KIT-b, KIT-c, and KIT-d (Table S1). Binding affinities of the variants to KIT protein were analyzed with SPR. Results showed that KIT-d exhibited the lowest Kd value (Figure S2). The serum stability of the aptamers was further studied and a similar half-life was detected (Figure S3). The binding ability of variants to GIST cells was further detected by flow cytometry (Figure 2A-B). Results show that KIT-d exhibited the best affinity, surpassing even that of KIT-w, while KIT-a and KIT-b retained partial affinity. KIT-c, on the other hand, lost its affinity. So, KIT-d was chosen for subsequent research. The shift of KIT-d fluorescence was consistent with the results of the anti-KIT (Figure 1H), and the correlation between KIT-d and the anti-KIT antibody was 0.9243 (p < 0.0001) (Figure 2C). Confocal experiments showed that both KIT-d and the anti-KIT antibody were detected in KIT-positive cells, and significant colocalization was observed on the cell membrane (Figure 2D and Figure S4), confirmed the binding of KIT-d aptamer to KIT. In addition, the apparent dissociation constant (Kd) of KIT-d for GIST-T1, GIST-430, and GIST-430/654 cells were 76.0 nM, 41.6 nM, and 109.3 nM respectively, indicating a desirable binding affinity (Figure 2E).

By predicting the secondary structures of KIT-w and KIT-d, we identified a common stem-loop structure in both (Figure 2F). The truncated variant KIT-c lacks this sequence, leading us to speculate that this particular structure is critical for the binding of KIT-w and KIT-d to KIT. To further understand the binding model of KIT-d to the KIT protein, computational modeling analysis was performed. As shown in Figure 2G and Figure S5, 5 residues (ARG353, GLY470, ASN406, GLU435, and ASN463) of KIT bind with 5 bases of KIT-d (A18, G20, G21, G24, and A29), and these bases are consistent with the stem-loop structure depicted in Figure 2F. Subsequently, we introduced mutations into the stem-loop structure of KIT-d, which is predicted to interact with the KIT protein, as shown in Figure 2F. The mutated versions, KIT-d-mu1, KIT-d-mu2 and KIT-d-mu3, exhibited significantly reduced binding ability for GIST cells (Figure S6). Next, we assessed whether KIT-d could block KIT protein on cell membrane with a competition assay in GIST-T1 and GIST-430/654 cells. Specifically, the anti-KIT antibody used here targets amino acids 350-440 of the KIT protein, encompassing the binding site for KIT-d identified by our computational modeling analysis. Results reveal that KIT antibody pretreatment significantly reduced the binding of KIT-d to GIST cells (Figure 2H), and conversely, high concentrations of KIT-d pre-treatment also significantly diminished the binding of the KIT antibody to GIST cells (Figure 2I). Taken together, these results indicated that KIT-d could specifically target the KIT protein on cancer cells. Further, we assessed the Kd of KIT-d and KIT-w to KIT protein with different methods. Flow cytometry (80.2 nM vs 105.9 nM) (Figure 2J) and surface plasmon resonance (71.07 nM vs 84.00 nM) (Figure S2) revealed that KIT-d has a similar affinity to KIT compared to KIT-w.

### ApDC construction and characterization

**Figure 3:**
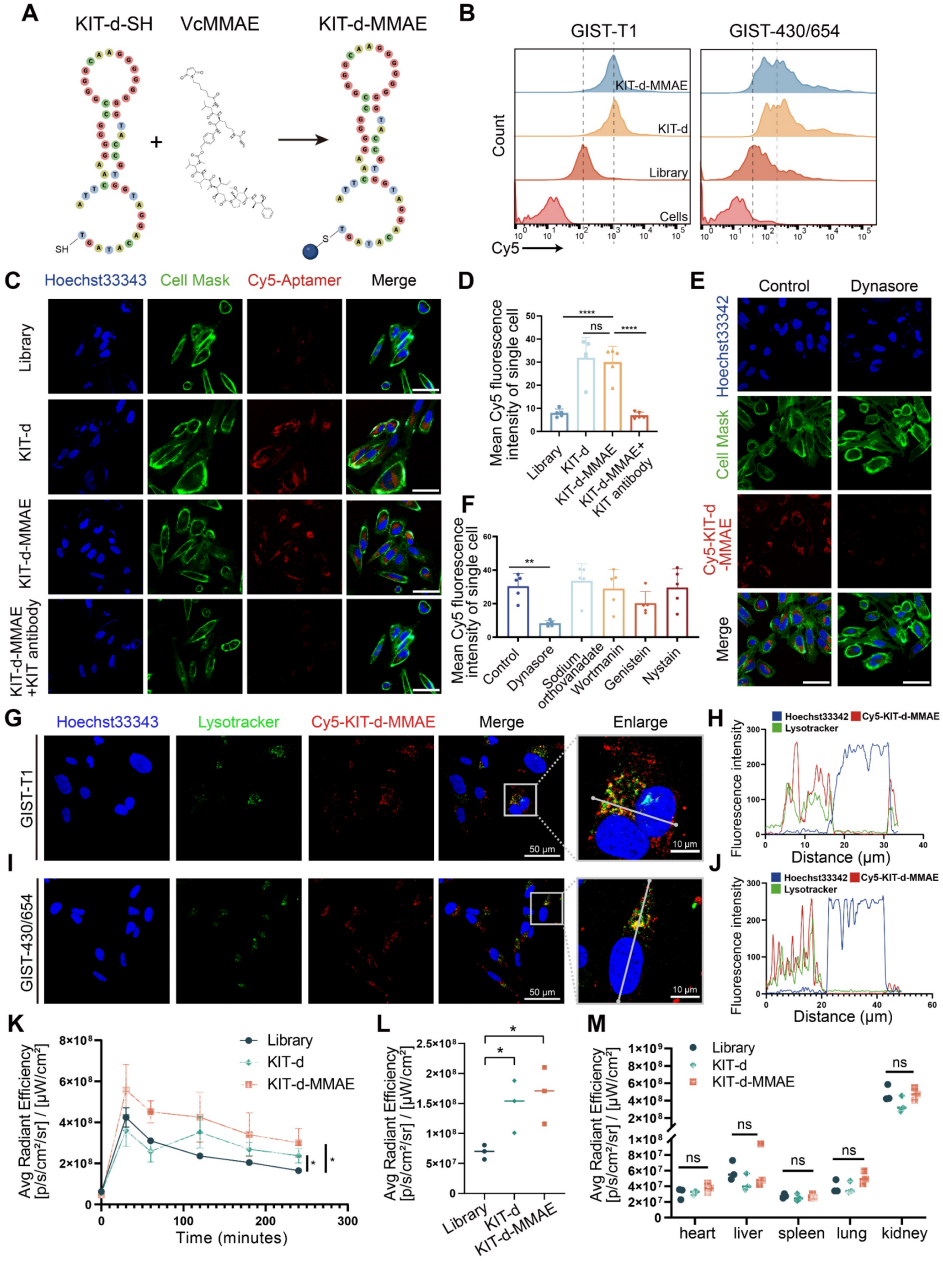
** ApDC Construction and Characterization.** (**A**) Schematic diagram of the construction of aptamer-drug conjugate (KIT-d-MMAE). (**B**) The binding affinity of KIT-d-MMAE (Cy5 labeled) for GIST-T1 and GIST 430/654 cells. (**C**) The internalization effect of aptamer and ApDC in GIST-430/654 cells were visualized after treatment with Cy5 conjugated KIT aptamers or pretreated with KIT blocking Abs. Hoechst33342 was used to counterstain the nuclei (blue). Cell Mask was used to counterstain the membrane (green). Scale bar = 50 μm. (**D**) Statistics of the Cy5 mean fluorescence intensity within individual cells in Figure 3C. (**E**) Live-cell imaging of GIST-430/654 cells after treatment with Cy5-KIT-d-MMAE or pretreated with the internalization inhibitor (Dynasore). Scale bar = 50 μm. (**F**) Statistics of the Cy5 mean fluorescence intensity of individual cells in Figure 3E and Figure S9. (**G-J**) Colocalization of Cy5-KIT-d-MMAE with Lysotracker Green in GIST-T1 and GIST-430/654 cells. The fluorescence intensity in single cells was quantified in the right chart. (**K**) Average fluorescence intensity of tumor sites in GIST-T1 tumor-bearing mice at 0, 0.5, 1, 2, 3, and 4 h after intravenously injection with 1 nmol Cy5-labeled library, KIT-d or KIT-d-MMAE related to Figure S10B. (**L**) Biodistribution of Cy5-Lib, Cy5-KIT-d, or Cy5-KIT-d-MMAE in the tumors at 4 h after injection related to Figure S10C. (**M**) Biodistribution of Cy5-Lib, Cy5-KIT-d, or Cy5-KIT-d-MMAE in the major organs at 4 h after injection related to Figure S10C. All data are presented as the mean ± SD. ns = not significant. **P < 0.01, ****P < 0.0001.

Then, we developed an ApDC by conjugating KIT-d with MMAE using a maleimide linker, a method widely used in antibody-MMAE conjugations (Figure 3A). The conjugated compound, KIT-d-MMAE, was validated and characterized using HPLC and mass spectrometry. Mass spectrometry confirmed the expected molecular weight of 15,977 Da, with the observed peak at 15,977.6 Da (Figure S7). HPLC analysis revealed a dominant peak at 8.935 min corresponding to KIT-d-MMAE, accounting for 95.6% of the total peak area, indicating a high purity of the conjugate (Figure S8). The binding ability of ApDC was first studied, compared to KIT-d, the KIT-d-MMAE conjugate retained the same binding ability against GIST-T1 and GIST-430/654 cells (Figure 3B). Next, confocal microscopy was utilized to evaluate the internalization potential of KIT-d-MMAE. As shown in Figure 3C, clear fluorescence was observed in KIT-d group compared to library group. Moreover, pretreatment with a KIT antibody significantly reduced the internalization of KIT-d-MMAE, suggesting its uptake is reliant on KIT (Figure 3D). To identify the potential endocytic pathways involved in KIT-d-MMAE internalization, five different endocytosis inhibitors were used to pre-treat GIST-T1 cells before incubation with KIT-d-MMAE. Results reveal that the adenosine triphosphatase inhibitor sodium orthovanadate, macropinocytosis inhibitor wortmannin, caveolae inhibitor genistein, and raft endocytosis inhibitor nystatin showed no significant impact, while the clathrin-dependent endocytosis inhibitor dynasore markedly reduced KIT-d-MMAE uptake (Figure 3E-F and Figure S9). To track the endolysosomal pathway, we performed microscopy experiments with Cy5-conjugated KIT-d-MMAE and lysotracker. Rapid colocalization of internalized KIT-d-MMAE with lysosomes was found and colocalization was further analyzed. Despite colocalization with lysosomes, a notable signal was also detected in the cytoplasm (Figure 3G-J), suggesting the release of drug.

To further explore the *in vivo* application potential, the tissue distribution of ApDC was studied in a xenografted model of GIST-T1 cells using *in vivo* imaging. As shown in Figure S10A, S10B and Figure 3K, a stronger fluorescence signal at the tumor area was detected after injection compared to the control group. Four hours later, the mice were euthanized, and the tumors along with major organs were harvested to assess the fluorescence intensity (Figure S10C). Results show that the kidneys exhibited the highest fluorescence intensity, reflecting the aptamer's primary excretion through the renal pathway. The KIT-d-MMAE and KIT-d groups showed significantly higher fluorescence intensity at the tumor site compared to the library group (Figure 3L), while fluorescence intensity showed no difference at major organs in the three groups (Figure 3M), suggesting the *in vivo* tumor targeting effect of ApDC and its application potential.

### Targeting cytotoxicity of KIT-d-MMAE to KIT-high and KIT-low GIST cell lines

**Figure 4:**
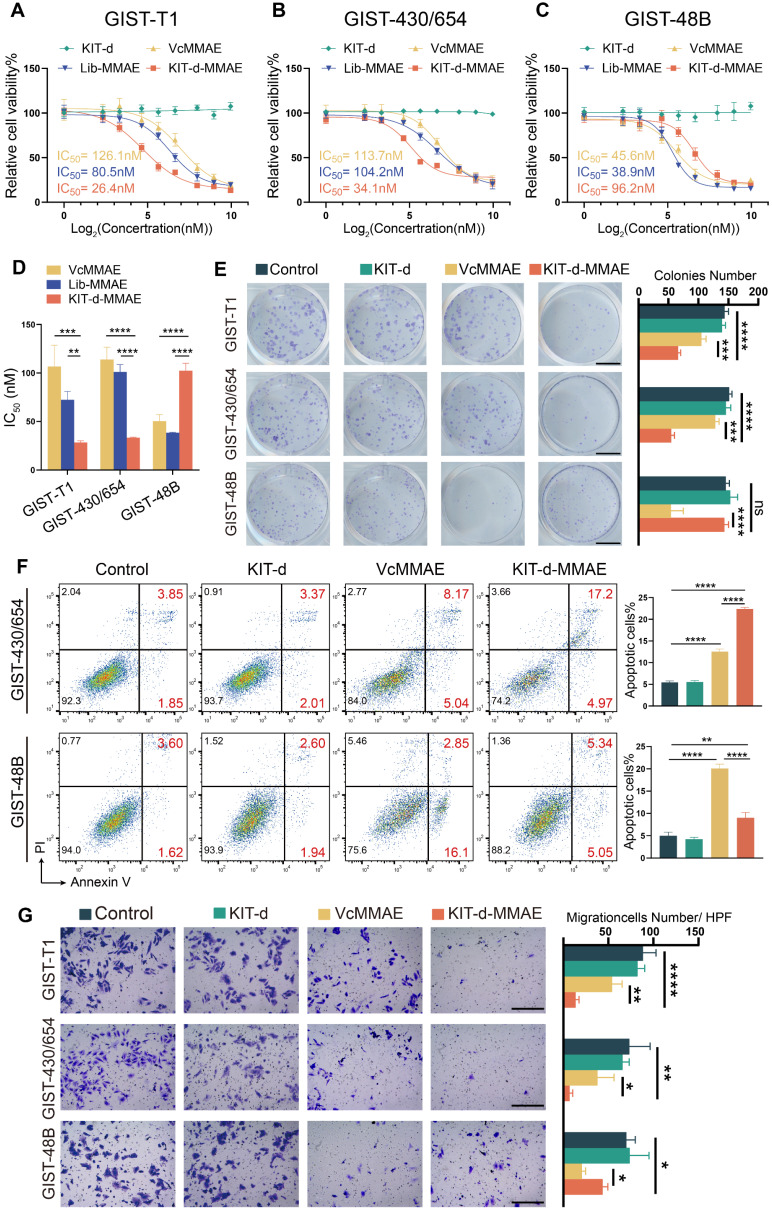
** Targeting cytotoxicity of KIT-d-MMAE to GIST cell lines.** (**A-C**) Cell viability of GIST-T1, GIST430/654, and GIST-48B cells with KIT-d, VcMMAE, Lib-MMAE or KIT-d-MMAE treatment. IC_50_ values of VcMMAE (yellow), Lib-MMAE (blue) and Kid-d-MMAE (red) are calculated and shown in the chart. (**D**) Statistical analysis and comparison of IC_50_ values in Figure 4A-C. (**E**) Colony formation of GIST-T1, GIST 430/654 and GIST-48B with the treatments of drugs. Scale bar = 10 mm Quantification results of the colony numbers were shown in the right histograms. (**F**) Apoptotic of GIST430/654 and GIST-48B cells with the treatments of drugs were studied with flow cytometry, the apoptosis ratio was shown. (**G**) The migration ability of GIST-T1, GIST430/654 and GIST-48B cells with the treatment of drugs was detected with the trans-well assay. Scale bar = 300 μm. Quantification results of the migration cell numbers were shown in the right histograms. All data are presented as the mean ± SD. ns = not significant. *P < 0.05, **P < 0.01, ***P < 0.001, ****P < 0.0001.

Cytotoxicity was important for drug application, thus the IC50 of ApDC was first studied (Figure 4A-C). Since *KIT* is a driver gene in the development of GIST, knocking down or out *KIT* significantly affects cell proliferation, posing challenges in constructing stable transgenic cell lines 39. Therefore, KIT-negative GIST-48B cells was chosen as the negative control, and GIST-T1 and GIST-430/654 cells were chosen as positive cells. Results show that KIT-d-MMAE demonstrated notably lower IC50 values than VcMMAE and Lib-MMAE in GIST-T1 and GIST-430/654, but higher IC50 values in GIST-48B (Figure 4D). The antiproliferative potential of KIT-d-MMAE was further verified. Cells were incubated with KIT-d, VcMMAE, and KIT-d-MMAE at a concentration of 100 nM. As shown in Figure S11, KIT-d aptamer did not influence cell viability, while KIT-d-MMAE exhibited significant inhibitory effects on cell viability compared to VcMMAE in GIST-T1 and GIST-430/654 cell lines. In contrast, in the KIT-negative GIST-48B cell line, the inhibitory efficacy of ApDC was notably weaker than VcMMAE. Similar results were observed for the colony formation (Figure 4E).

As a microtubule inhibitor, MMAE exerts its effect by inhibiting cell mitosis, resulting in cell cycle arrest at the G2/M phase^40^. The effect of KIT-d-MMAE on cell cycle was first investigated. As shown in Figure S12 and S13, in GIST-T1 and GIST-430/654 cells, KIT-d-MMAE induced a significantly stronger cell cycle arrest compared to VcMMAE, whereas in the GIST-48B cells, its effect was less pronounced than VcMMAE. Cell cycle arrest always resulted cell apoptosis, so apoptotic cells were analyzed by flow cytometry. We found that treatment with KIT-d-MMAE increased apoptotic cells in GIST-T1 and GIST-430/654 cells, while the opposite result was observed in GIST-48B cells (Figure 4F, Figure S14 and S15). TUNEL assay results also demonstrated that KIT-d-MMAE induces markedly greater apoptosis in GIST-T1 cells than VcMMAE (Figure S16). Inspect of cell proliferation and cell cycle, tubulin was also associated with invasion and migration, thus the effect of ApDC on migration was tested with transwell assay (Figure 4G). Consistent with above results, decreased migration cells were observed in ApDC group compared to VcMMAE. The phosphorylation levels of KIT and its downstream signaling molecules AKT and MAPK were studied in GIST-T1 cells following KIT-d-MMAE treatment. The results indicate that KIT-d-MMAE does not significantly alter the expression levels of p-KIT, p-AKT, or p-MAPK (Figure S17). Unlike TKIs that inhibit KIT phosphorylation by binding to ATP, KIT-d-MMAE binds to the extracellular domain of KIT and is internalized into the cell, leading to drug release and tumor cell apoptosis.

These findings collectively indicate that KIT-d-MMAE induces cell cycle arrest, leading to stronger antiproliferative and anti-migration effects compared to VcMMAE in KIT-positive tumor cells, not by suppressing KIT signaling activity, but rather through targeted drug delivery and intracellular cytotoxicity.

### Pharmacokinetics and distribution evaluation of KIT-d-MMAE

**Figure 5:**
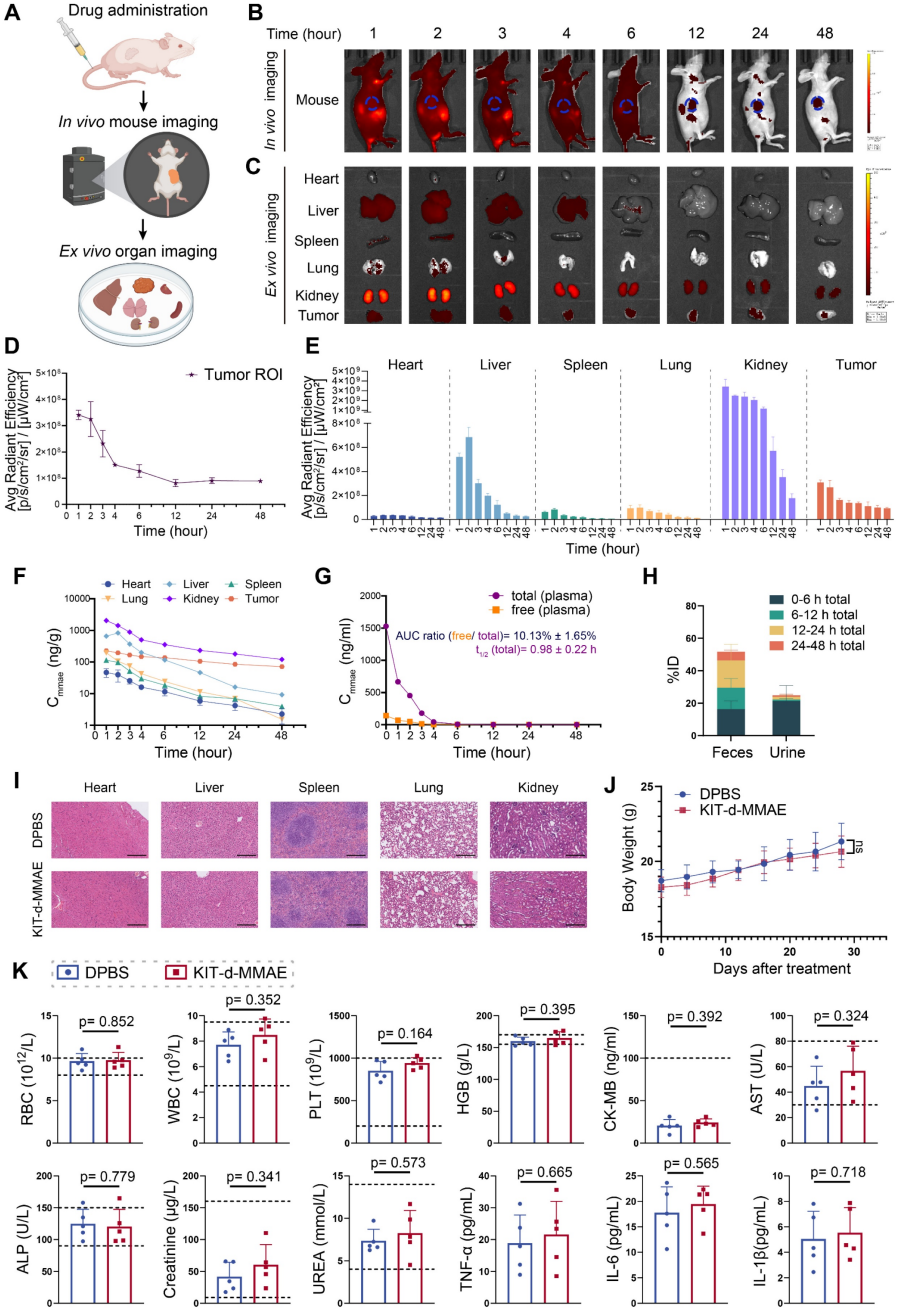
** Pharmacokinetics and biosafety evaluation of KIT-d-MMAE.** (**A-E**) *In vivo* imaging of KIT-d-MMAE in GIST-T1 tumor-bearing nude mice. (**A**) Schematic illustration of the experimental workflow. **(B)** IVIS images at the indicated time points following tail vein injection of 7.5 nmol Cy5-labeled KIT-d-MMAE (n = 3). (**C**) *Ex vivo* imaging of major organs and tumors at corresponding time points. (**D**) Quantification of tumor fluorescence intensity from Figure 5B. (**E**) Quantification of organ fluorescence intensity from Figure 5C. (**F-H**) Pharmacokinetics and metabolic distribution of KIT-d-MMAE. (**F**) MMAE concentrations in major organs and tumors at various time points post-injection. (**G**) Plasma levels of total and cleaved free MMAE over time; the AUC ratio of free MMAE, as well as the half-life and clearance rate, was calculated. (**H**) Cumulative drug excretion profile after intravenous administration. (**I-K**) Biosafety evaluation of KIT-d-MMAE. (**I**) H&E staining of major organs after six doses of KIT-d-MMAE over 28 days in GIST-T1 tumor-bearing mice. Scale bars = 300 μm. (**J**) Body weight of mice was monitored throughout the treatment period. (**K**) Hematological, biochemical, and immunological parameters were measured after treatment, including RBC, WBC, PLT, HGB (hematology); CK-MB, AST, ALP, creatinine, UREA (biochemistry); and TNF-α, IL-6, IL-1β (cytokines). All data are presented as the mean ± SD. ns = not significant.

To characterize the pharmacokinetics and biodistribution of KIT-d-MMAE, we performed *in vivo* and *ex vivo* imaging, along with LC-MS/MS-based quantification of MMAE in plasma, tumors, and major organs. Following intravenous injection of ApDC in GIST-T1 tumor-bearing mice, strong fluorescence signals were observed at the tumor site within 1 h and remained detectable for up to 48 h (Figure 5B and 5D), indicating rapid accumulation and prolonged tumor retention. *Ex vivo* imaging further revealed low and transient signals in the heart, spleen, and lungs, moderate uptake in the liver, and high but gradually decreasing intensity in the kidneys (Figure 5C and 5E). The fluorescence signal persisted beyond 12 h in tumor, indicating sustained intratumoral accumulation (Figure 5E).

These imaging results were corroborated by LC-MS/MS measurements of MMAE levels. The kidneys exhibited the highest drug exposure within 24 h, followed by the liver, while drug concentrations in most non-tumor tissues declined markedly by 12 h. In contrast, the tumor retained high MMAE levels over time (Figure 5F). Plasma pharmacokinetics showed rapid clearance of KIT-d-MMAE, with a half-life of 0.98 h. The AUC ratio of cleaved free MMAE was approximately 10%, suggesting good *in vivo* stability and minimal premature drug release (Figure 5G). Excretion analysis revealed that over 70% of the administered dose was eliminated within 24 h, predominantly via feces (~50%) and to a lesser extent via urine (~20%), with early-phase clearance driven by renal excretion and late-phase by fecal elimination (Figure 5H).

Together, these findings demonstrate that KIT-d-MMAE preferentially accumulates and persists in tumor tissue, while being rapidly cleared from circulation and normal organs, suggesting its potential for effective and low-toxicity targeted therapy.

### Biosafety evaluation of KIT-d-MMAE

To comprehensively assess the *in vivo* biosafety of KIT-d-MMAE, we conducted toxicity evaluations in both ICR mice and GIST-T1 tumor-bearing nude mice under different dosing regimens^41^. ICR mice were administered 4 mg/kg of KIT-d-MMAE via tail vein injection and analyzed at 1-, 14-, and 28-days post-treatment to assess acute, intermediate, and chronic effects (Figure S18 and S19). In parallel, tumor-bearing mice received repeated doses of KIT-d-MMAE (4 mg/kg) to evaluate long-term toxicity in a therapeutic context (Figure 5I-K). In both models, histological examination of major organs by H&E staining revealed no observable tissue abnormalities (Figure 5I, Figure S18). Body weight remained stable across all treatment groups, with no significant differences compared to DPBS group throughout the study period, indicating the biosafety of KIT-d-MMAE (Figure 5J, Figure S19).

Comprehensive blood analyses showed no significant changes in key parameters, including hematological indices (RBC, WBC, PLT, HGB) and biochemical markers (CK-MB, AST, ALP, creatinine, urea) (Figure 5K, Figure S19). Although ALT and AST levels were mildly elevated in the ICR mice at day 1, the changes were not statistically significant and returned to baseline at later time points, suggesting minimal hepatotoxicity.

Notably, despite the high renal accumulation of KIT-d-MMAE observed in imaging and distribution studies, no signs of nephrotoxicity were detected. Histological analysis of kidney tissue showed no pathological alterations (Figure 5I, Figure S18), and serum creatinine and urea levels remained within normal physiological ranges, indicating preserved renal function (Figure 5K, Figure S19). In addition, cytokine profiling (TNF-α, IL-6, IL-1β) showed no elevation in inflammatory markers, further supporting the low immunogenicity of KIT-d-MMAE (Figure 5K).

Collectively, these findings demonstrate that KIT-d-MMAE has a favorable biosafety profile across animal models and dosing regimens, with no evidence of hepatic, renal, hematologic, or immunologic toxicity.

### Antitumor activity of ApDC in xenograft models

**Figure 6:**
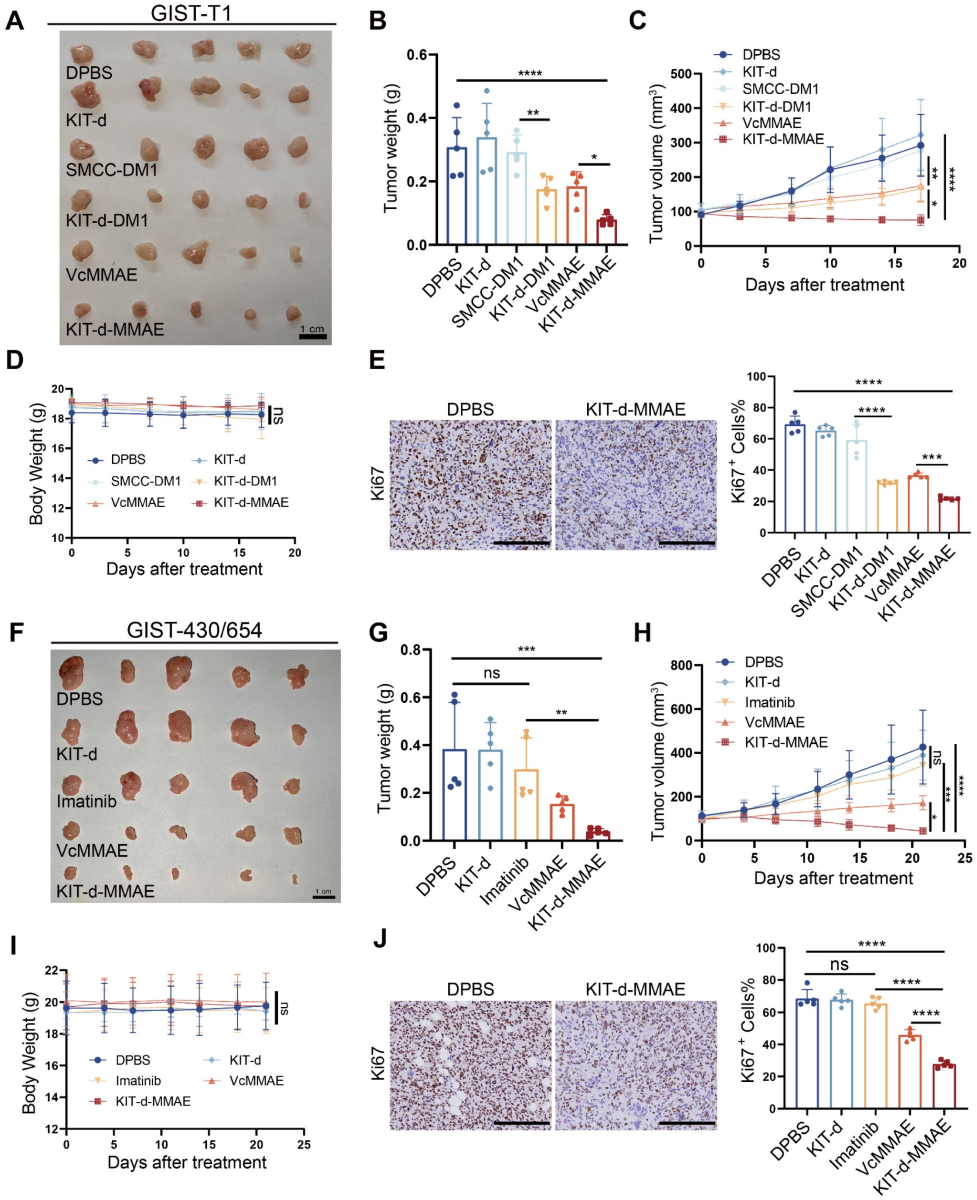
** Targeted therapy effect of ApDC in xenograft models.** (**A-E**) Antitumor activity of ApDC in GIST-T1 xenograft model (5 mice/group). (**A**) Images of tumor tissues from mice sacrificed at 17 days after treatment. (**B**) Tumor weight of mice after treatment. (**C**) Growth curve of the tumors during therapy. (**D**) Mice body weight was recorded during therapy. (**E**) Ki67 staining images of tumor sections (left) and corresponding statistical charts of Figure S22 (right). Scale bar = 200 μm. (**F-J**) Antitumor effect of KIT-d-MMAE in GIST-430/654 (imatinib-resistant) xenograft model (5 mice/group). (**F**) Images of tumor tissues from mice sacrificed at 21 days after treatment. (**G**) Tumor weight of mice after treatment. (**H**) Growth curve of the tumors during therapy. (**I**) Mice body weight was recorded during therapy. (**J**) Ki67 staining images of tumor sections (left) and corresponding statistical charts of Figure S23 (right). Scale bar = 200 μm. All data are presented as the mean ± SD. ns = not significant. *P < 0.05, **P < 0.01, ***P < 0.001, ****P < 0.0001.

To evaluate the antitumor effect of KIT-d-MMAE *in vivo*, an orthotopic cell line-derived xenograft (CDX) model was established using human GIST-T1 cells (Figure 6A-E). Previous research indicated effective treatment outcomes with DM1-conjugated ADCs 20, so we also synthesized a KIT-d-DM1 conjugated ApDC for in *vitro* and *in vivo* anti-tumor assessment. As shown in Figure S20, KIT-d-DM1 demonstrated targeted cytotoxicity towards GIST cell lines just like KIT-d-MMAE. *In vivo* therapy effect of KIT-d-MMAE and KIT-d-DM1 were studied when the tumor volume reached 80-120 mm^3^: mice were randomized into six groups (n = 5) and injected with DPBS, KIT-d, SMCC-DM1, KIT-d-DM1, VcMMAE or KIT-d-MMAE via tail vein twice a week for 5 times. Animals treated with KIT-d-DM1, VcMMAE, and KIT-d-MMAE exhibited notably reduced tumor burden and slower tumor growth (Figure 6A-C). Additionally, the tumor-suppressive effects of KIT-d-MMAE and KIT-d-DM1 were distinctly superior to those of VcMMAE and SMCC-DM1, respectively, indicating that conjugation with the aptamer KIT-d enhances the targeted drug delivery efficacy. Moreover, KIT-d-MMAE revealed better antitumor activity compared to KIT-d-DM1. Therefore, KIT-d-MMAE was chosen for subsequent studies. Tumor homogenate was studied by Mass spectrometry to identify the concentration of MMAE. Results showed that the concentration of MMAE in KIT-d-MMAE group was about 3 times higher than that in VcMMAE group (Figure S21), suggesting a targeted delivery effect of aptamer. Then tumor sections were prepared and cell proliferation was studied with Ki67 stain. KIT-d-MMAE group displayed less intense Ki67 antigen staining, signifying a stronger anti-tumor effect (Figure 6E, Figure S22).

Next, we established another subcutaneous tumor transplantation model with the imatinib-resistant GIST-430/654 cell line (Figure 6F-J). Results show that limited tumor suppression was observed in imatinib group (about 22% inhibition rate, p = 0.27), proving the success construction of the mice model, while KIT-d-MMAE significantly curtailed tumor growth (approximately 89% inhibition rate, p = 0.0002), outperforming both imatinib and VcMMAE (Figure 6F-H). Similarly, the KIT-d-MMAE group exhibited a significant decrease in cell proliferation activity (Figure 6J and Figure S23). Notably, in both models, mice treated with KIT-d-MMAE did not show significant weight loss (Figure 6D and 6I). H&E staining of relevant organs also indicated no apparent organ pathology (Figure S24 and S25), consistent with the biosafety results, suggesting the *in vivo* application potential of KIT-d-MMAE.

To further assess the therapeutic potential of KIT-d-MMAE, we compared its antitumor efficacy with that of DS-6157a, a GPR20-targeted antibody-drug conjugate (ADC) that has been evaluated in preclinical studies and entered a Phase I clinical trial for GIST^19^, ^42^. The results showed that both agents effectively suppressed tumor growth at equivalent doses. However, KIT-d-MMAE showed greater tumor inhibitory activity than DS-6157a, highlighting its promise as a targeted therapeutic candidate for GIST (Figure S26).

### Targeted therapy effect of ApDC in PDX model

**Figure 7:**
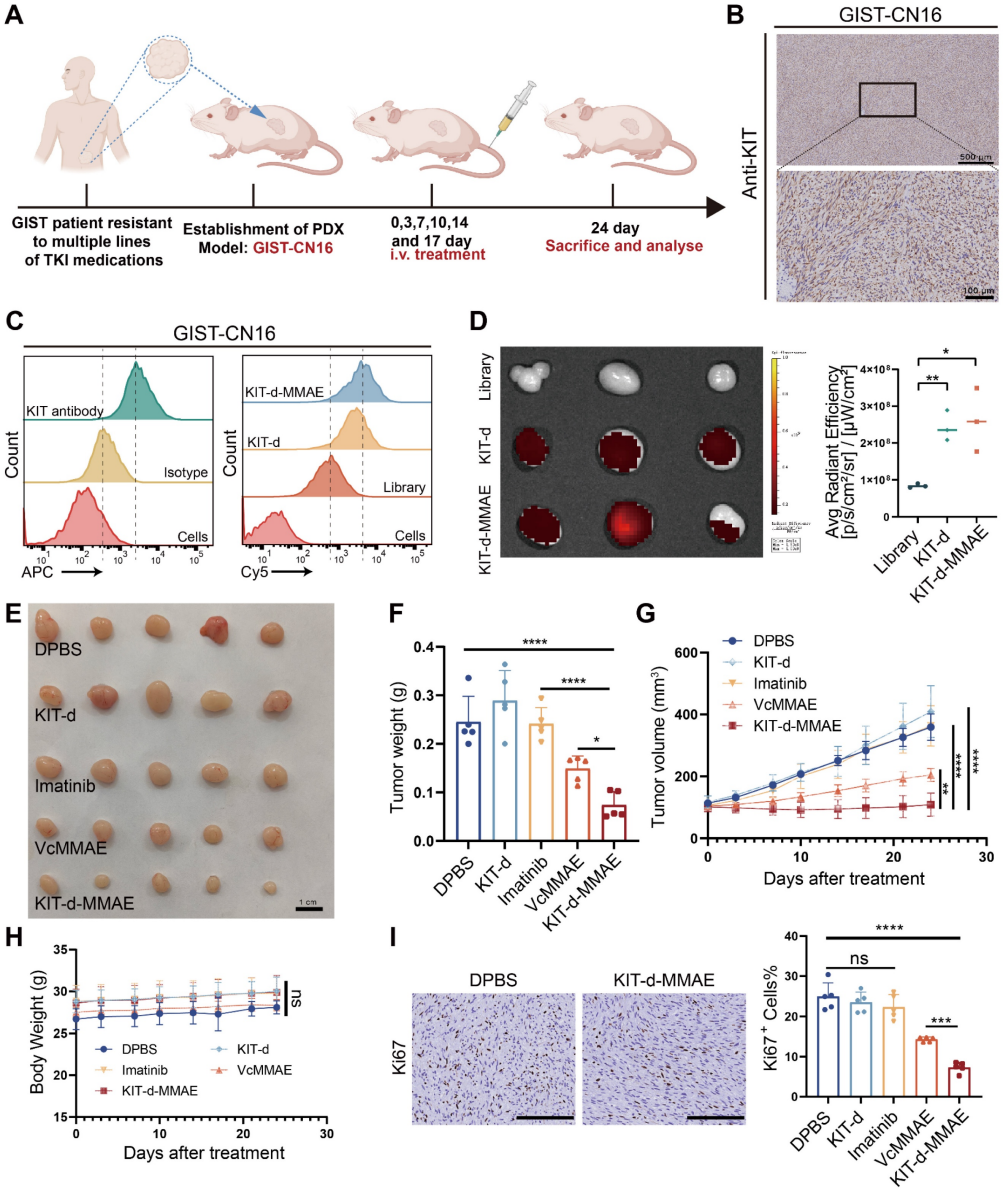
** Targeted therapy effect of ApDC in multiple TKI resistant PDX model.** (**A**) Schematic diagram of the construction of the PDX model (imatinib-resistant) and the treatment time. (**B**) Immunohistochemical staining of KIT in tumor sections from GIST-CN16. (**C**) The binding affinity of KIT antibody (APC labeled) and KIT aptamer (Cy5 labeled) to GIST-CN16 cells. (**D**) *Ex vivo* IVIS Lumina imaging of tumors at 4 h after the injection of the library, KIT-d or KIT-d-MMAE (left), and corresponding statistical charts (right). (**E**) Images of tumor tissues from mice sacrificed at 24 days after treatment. (**F**) Tumor weight of mice after treatment. (**G**) Growth curve of the tumors during therapy. (**H**) Mice body weight was recorded during therapy. (**I**) Ki67 staining images of tumor sections (left) and corresponding statistical charts of Figure S30 (right). Scale bar = 200 μm. All data are presented as the mean ± SD. ns = not significant. *P < 0.05, **P < 0.01, ***P < 0.001, ****P < 0.0001.

Currently, the standard treatment for GIST includes four lines of drugs: imatinib, sunitinib, regorafenib, and ripretinib. Once resistance to these four lines of medication develops, patients face a situation with no available treatment options 39. To explore the potential application of KIT-d-MMAE in TKI-resistant patients, we developed a GIST Patient-Derived Xenograft (PDX) model resistant to multiple TKIs (Figure S27). We also isolated and extracted primary tumor cells from this PDX, termed GIST-CN16. IHC and flow cytometry confirmed high KIT levels in GIST-CN16, and both KIT-d and KIT-d-MMAE showed strong affinity to GIST-CN16 (Figure 7B-C). LSCM showed effective internalization of KIT-d and KIT-d-MMAE into GIST-CN16 cells, while this internalization was significantly reduced by dynasore or anti-KIT antibody, aligning with our previous findings in Figure 3C and 3E (Figure S28 and S29). *Ex vivo* imaging of tumors from mice injected with Cy5 labeled aptamers also indicated that KIT-d and KIT-d-MMAE have a robust capacity for *in vivo* tumor targeting (Figure 7D). These findings are consistent with our previous experimental data, further substantiating the potential of KIT-d-MMAE for targeting patient-derived GIST. Subsequently, the established PDX model was treated with the drug, as depicted in Figure 7A, to evaluate the anti-tumor efficacy of KIT-d-MMAE. Contrary to expectations, imatinib showed minimal tumor suppression (about 1% inhibition rate, p = 0.8844), while KIT-d-MMAE significantly inhibited tumor growth (about 70% inhibition rate, p < 0.0001), outperforming both imatinib and VcMMAE (Figure 7E-G). Besides, the KIT-d-MMAE group exhibited a lower Ki67 positivity compared to other groups, suggesting reduced proliferation of tumor cells (Figure 7I and Figure S30). In all groups, no significant reduction in mice body weight was observed (Figure 7H), and H&E staining of major organs revealed no apparent tissue damage (Figure S31). These results demonstrated the targeting therapy ability of KIT-d-MMAE.

### Antitumor activity of KIT-d-MMAE in GIST spontaneous tumorigenesis model and liver metastasis model

**Figure 8:**
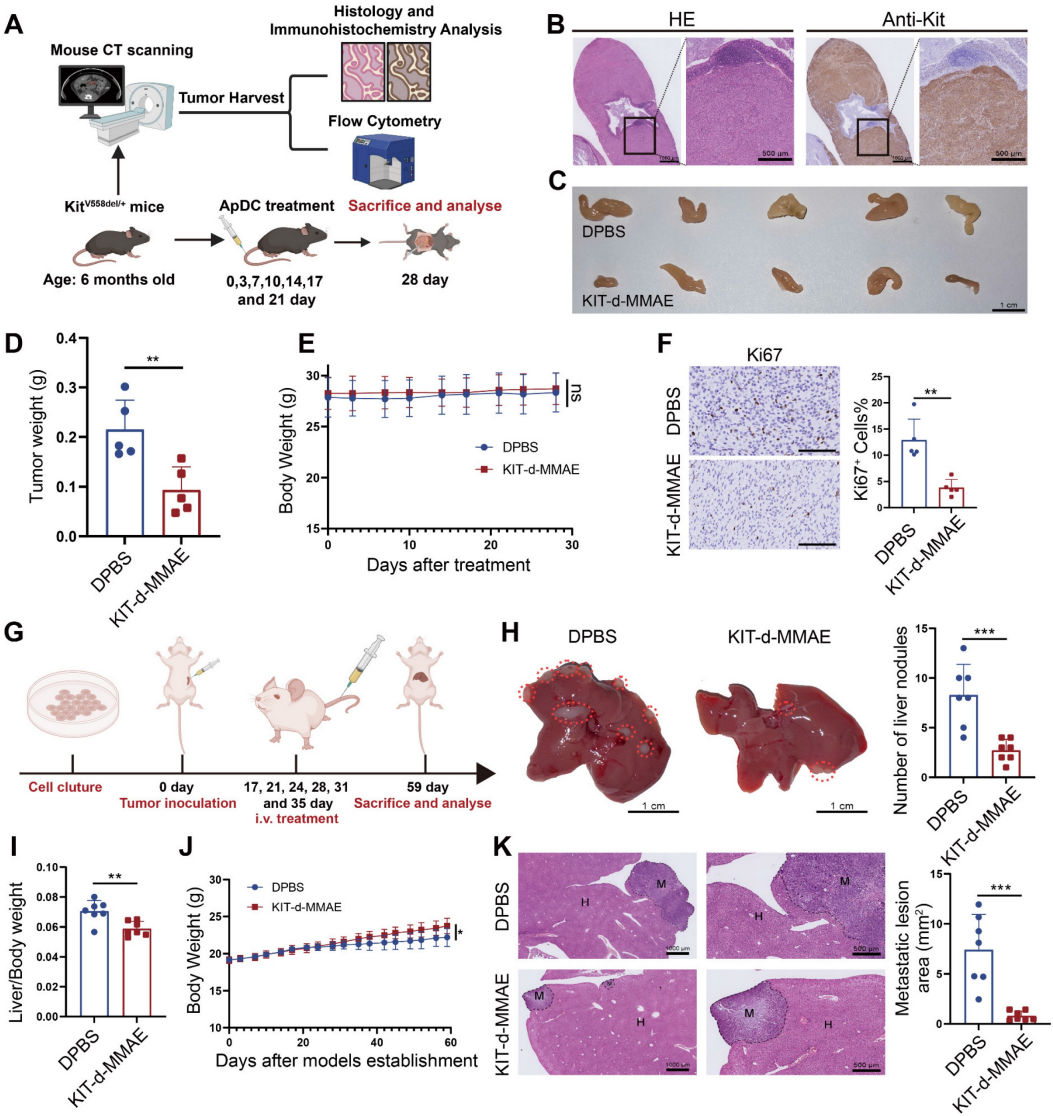
**Antitumor activity of KIT-d-MMAE in GIST spontaneous tumorigenesis model and liver metastasis model.** (**A-F**) Antitumor activities of KIT-d-MMAE in a GIST spontaneous tumorigenesis model (5 mice per group). (**A**) Schematic diagram of mice model construction and the treatment time. (**B**) Immunohistochemical staining of KIT in tumors from GIST spontaneous tumorigenesis model. (**C**) Images of tumor tissues from mice sacrificed at 28 days after treatment. (**D**) Tumor weight of mice after treatment. (**E**) Mice body weight was recorded during therapy. (**F**) Representative Ki67 staining images of tumor sections (left) and corresponding statistical charts (right). Scale bar = 100 μm. (**G-K**) Antitumor effect of KIT-d-MMAE in a GIST liver metastasis model (7 mice per group). (**G**) Schematic diagram of construction of liver metastasis model and treatment time. (**H**) Images of livers excised from mice bearing GIST-T1 cells after treatment with ApDC (left) and the metastasis nodule number was assessed (right). (**I**) Liver/ Body weight ratio in the above mice at the end of the treatments. (**J**) Mice body weight was recorded during therapy. (**K**) Representative images of H&E-stained liver sections (left) and quantification of metastatic lesion area (right). All data are presented as the mean ± SD. ns = not significant. *P < 0.05, **P < 0.01, ***P < 0.001.

Human and murine KIT exhibit 90% sequence homology, the predicted binding sites depicted in Figure 2G show complete concordance across both species, suggesting KIT-d may also bind with murine KIT. To further confirm its application potential a spontaneous tumorigenesis model was used: *Kit*^V558del/+^ mice model is a spontaneous tumorigenic model for GIST, capable of developing stromal tumors in the ileocecal region of the intestine around the age of three months, accurately representing GIST progression 33. High-resolution CT scans of the mice indicated tumor formation by the age of six months (Figure S32), then the mice were humanely euthanized and prominent tumor formations were observed in the ileocecal region. Immunohistochemical analysis for Kit expression in this region revealed strong positivity, further confirming the tissues as GIST (Figure 8B). We further dissociated the tissue to obtain a cell suspension and assessed the affinity of KIT-d for these cells. The results demonstrated that KIT-d also exhibits substantial affinity for spontaneously tumorigenic GIST cells, corroborating our earlier hypothesis that KIT-d may also bind with murine KIT (Figure S33). Then, we tested whether KIT-d-MMAE is also effective for this model. The mice were randomized into two groups and administered DPBS or KIT-d-MMAE as shown in Figure 8A. After 7 times treatment, mice were humanely euthanized to obtain the tumor (Figure 8C and Figure S34). The KIT-d-MMAE group demonstrated a substantial reduction in tumor weight, about 56% tumor inhibition rate (p = 0.0071) compared to the DPBS group (Figure 8D), along with significantly lower Ki67 levels (Figure 8F). Notably, the mice treated with KIT-d-MMAE showed no body weight loss (Figure 8E) or organ damage (Figure S35), further verifying that KIT-d-MMAE effectively inhibited tumor progress without any adverse effects.

Tumor metastasis is prevalent in advanced GIST, and liver metastasis is the most common (50-65%) 43. Given our earlier experimental data indicating the significant inhibitory effect of KIT-d-MMAE on tumor migration (Figure 4G), we further developed a GIST liver metastasis model with GIST-T1 cell to assess the potential of ApDC in tumor metastasis inhibition (Figure 8G). As illustrated in the gross liver images, KIT-d-MMAE significantly reduced the number of liver nodules (Figure 8H) and decreased the liver/body weight ratio, indicating a reduced burden of liver metastatic tumors (Figure 8I). Histological analysis indicated that the KIT-d-MMAE group had smaller areas of tumor metastasis and exhibited reduced Ki67 level in tumor cells, suggesting lower proliferation rates, compared to the DPBS group (Figure 8K and Figure S36). Mice in the KIT-d-MMAE group did not exhibit significant weight loss; conversely, the weight gain rate in the DPBS group was lower than in the KIT-d-MMAE group, possibly due to liver dysfunction caused by tumor metastasis (Figure 8J). Results above confirmed the antitumor activity of ApDC in GIST.

## Discussion and Conclusion

Surgical excision followed by Imatinib therapy is a standard approach for advanced GIST, although imatinib effectively suppresses tumors growth, it cannot fully eliminate tumor cells 11, 12. This necessitates prolonged treatment, often exceeding three years, which poses several challenges 39. First, imatinib is a broad-spectrum TKI, targeting not only KIT but also receptors like CSF1R and DDR1, potentially causing severe side effects and treatment discontinuation 44. Additionally, long-term therapy leads to a significant financial burden for patients. Most critically, dormant tumor cells may mutate over time, resulting in drug resistance 9, 10. Consequently, many patients develop resistance to imatinib, leading to tumor recurrence and adversely affecting survival. Therefore, more specific targeting drugs need to be developed. Aptamers as “Chemical antibody”, exhibit promising targeting effect *in vitro* and *in vivo*, aptamer drug conjugates have been reported effectively in different tumor models 28.

Prior studies have identified a KIT-targeting nucleic acid aptamer with therapeutic potential in AML 38, and Ray P further demonstrated its ability to target GIST through *in vitro* and *in vivo* imaging 45, 46. These important findings established the foundation for KIT-targeted strategies in GIST. Besides the diagnostic or imaging applications of KIT-targeting aptamer, we verified its therapy and clinical application potential in GIST. First, the aptamer was truncated to reduce synthesis cost for commercial process, mutation sequences and molecular docking were performed to confirm the binding site between aptamer and KIT. Then, the tubulin inhibitor MMAE was conjugated to aptamer with a VC linker to construct ApDC, which kept the binding affinity of aptamer and the cytotoxicity of MMAE, so as to target and kill KIT positive cells. The efficacy, pharmacokinetics, *in vivo* stability, and biosafety of the ApDC were systematically evaluated. Moreover, the therapeutic potential of ApDC was confirmed in multiple clinically relevant models, including imatinib-sensitive, imatinib-resistant, and multi-TKI-resistant GISTs. The therapeutic efficiency was also compared with other payloads such as KIT-d-DM1, ADC and TKI. Compared with KIT-d-DM1, ADC, second- and third-line therapies such as sunitinib and regorafenib, which offer limited progression-free survival (6-8 months) and are often associated with significant toxicity 47, KIT-d-MMAE demonstrated superior efficacy and safety.

First, KIT-d-MMAE exhibited promising anti-tumor activity in GIST animal models, including those resistant to imatinib. The strong antitumor effects in GIST-T1 subcutaneous and liver metastasis models confirmed KIT-d-MMAE's efficacy in suppressing tumor growth and metastasis in advanced GIST, underscoring its potential as a powerful treatment, especially for prevalent liver metastases in advanced stages. Moreover, imatinib-resistant GIST-430/654 and multi-TKI-resistant PDX models, where KIT-d-MMAE showed promising antitumor while imatinib had little effects, suggesting its utility as an alternative therapy for TKI-resistant GIST. Additionally, antitumor activities were observed in genetically engineered mouse models, suggest effective inhibition of *in situ* GIST.

Biosafety is a key consideration for clinical translation. KIT-d-MMAE exhibited a favorable safety profile both *in vitro* and *in vivo*. In KIT-negative GIST-48B cells, its cytotoxicity was significantly reduced compared to free MMAE, indicating lower off-target toxicity due to aptamer-guided delivery. In multiple tumor models, KIT-d-MMAE treatment resulted in effective tumor suppression without noticeable weight loss or organ damage. Recent pharmacokinetic and toxicity data further support its safety. Despite high renal distribution, no signs of nephrotoxicity were observed. Repeated dosing in both healthy and tumor-bearing mice showed no significant alterations in body weight, histology, hematological, biochemical, or inflammatory parameters. These findings collectively confirm the biosafety of KIT-d-MMAE and support its potential for clinical development.

Simultaneously, several advantages were observed when compared KIT-d-MMAE with imatinib. 1) KIT-d-MMAE specifically targets KIT-positive cells, whereas imatinib has a broader targeting spectrum, implying that off-target toxicity is significantly reduced with KIT-d-MMAE. 2) Compared to imatinib, which only inhibits tumor proliferation, KIT-d-MMAE significantly induces apoptosis in GIST cells. This suggests that KIT-d-MMAE has the potential to eradicate residual GIST lesions in the body, reducing the risk of drug resistance caused by the remaining dormant tumor cells. 3) In our studies involving imatinib-resistant cells and animal models, KIT-d-MMAE demonstrated significant anti-tumor efficacy. This implies that patients clinically resistant to imatinib may also benefit from KIT-d-MMAE treatment, transcending mutation types.

In summary, our study introduces a promising and innovative therapeutic approach with the potential to substantially influence GIST treatment. Particularly for those who have developed resistance to imatinib, this medication presents an alternative therapeutic approach. It also holds the potential for treating various KIT-expressing malignancies, including melanomas, acute myeloid leukemia (AML), seminomas, small-cell lung cancer, and prostate cancer 48. This novel approach offers persuasive preclinical evidence warranting additional translational research.

## Supplementary Material

Supplementary methods, table and figures.
