# Supplementary material for: Aptamer-based drug delivery for targeted therapy of imatinib-resistant gastrointestinal stromal tumor
Source: Theranostics. 2025 Aug 11;15(17):8738–56. doi: 10.7150/thno.115496 (PMC12439145; doi:10.7150/thno.115496)
Supplement: Supplementary file 1 — Supplementary methods, table and figures. [file thnov15p8738s1.pdf]

Supplementary Information

Aptamer-based drug delivery for targeted therapy of imatinib-resistant gastrointestinal stromal tumor

Tao Pan [a,1], Ming Wang [a,1], Linxi Yang [a,1], Yihan Zheng [a,1], Yuanding Liu [a], Yu Xiao [a], Xudong Qiu [a], Yanying Shen [a], Mahan Dawuren [a], Zhiqiang Ren [a], Keying Liu [a], Yang Sun [a, *], Lin Tu [a, *], Hui Cao [a, *], Weihong Tan [a, b,*]

[a] Department of Gastrointestinal Surgery, Institute of Molecular Medicine (IMM), Department of Pathology, Ren Ji Hospital, Shanghai Jiao Tong University School of Medicine, Shanghai 200127, China

[b] Zhejiang Cancer Hospital, Hangzhou Institute of Medicine (HIM), Chinese Academy of Sciences, Hangzhou 310022, China

* Corresponding authors.

E-mails: suny1989@sjtu.edu.cn (Y. Sun), tulin@renji.com (L. Tu), caohuishcn@hotmail.com (H. Cao), tan@hnu.edu.cn (W. Tan)

[1] These authors contributed equally to this work.

Supplementary Methods

Cell Culture

Human GIST cell lines GIST-T1 was purchased from Cosmo Bio (Japan), while GIST-430, GIST-430/654, and GIST-48B were kind gifts from Prof. Jonathan Fletcher (Brigham and Women's Hospital, Harvard Medical School, USA). These four cell lines were cultured in Iscove's Modified Dulbecco's Medium (IMDM) (Gibco) with 10% fetal bovine serum (FBS) (Gibco) and 1% antibiotic mixture (Gibco) at 37°C with 5% CO_2. HEK293T, BXPC-3, MIAPaCa-2, PANC-1, and PATU8988T were purchased from the American Type Culture Collection (ATCC) and cultured in RPMI-1640 medium (Gibco) with 10% FBS and 1% antibiotic mixture at 37°C with 5% CO_2. GIST-CN16 was a primary cell line derived from tumors of patients with GIST who exhibit resistance to TKIs and was cultured in IMDM with 20% FBS and 1% antibiotic mixture at 37°C with 5% CO_2.

Reagents

The related chemical products are summarized below:

MC-Val-Cit-PAB-MMAE (HY-15575, MCE, USA); SMCC-DM1 (HY-101070, MCE, USA); Imatinib (HY-50946, MCE, USA); Tris(2-carboxyethyl) phosphine hydrochloride (TCEP, Sigma Aldrich, USA); All oligonucleotides' sequences (shown in Tables S1) were synthesized and purified by Sangon Biotech Co., Ltd. (Shanghai, China).

Animals

All animals were maintained in a pathogen-free environment at the Renji Hospital

(Shanghai, China) animal facility with controlled temperature, humidity, and light-dark cycle. All animal studies were reviewed and approved by the Ethics Committee of Renji Hospital (2018-029, RJ-2023-114A) and were performed by NIH guidelines.

Immunohistochemistry (IHC)

TMAs and bulk tissue specimens were first subjected to deparaffinization using xylene, followed by a graded alcohol hydration series. Then, the endogenous peroxidases were eliminated by 0.3% H_2O_2 at 37°C for 20 minutes. For antigen blocking, a 10% solution of bovine serum albumin (BSA) was employed for 60 minutes. This was followed by the application of primary antibodies, which were incubated at 4°C through the night. Thereafter, slides were treated with horseradish peroxidase (HRP)-linked secondary antibodies for 60 minutes at room temperature, and visualization was achieved using a DAB substrate kit (8059, Cell Signaling Technology). Before imaging with Leica microscopy, the tissues were counterstained with hematoxylin. For the quantification of KIT protein expression, a four-tiered classification system was employed: negative, weak, moderate, and high expression, determined based on both the intensity of staining and the extent of positive area coverage.

Antibodies for IHC contained: c-KIT (37805, CST, USA, 1:400), c-Kit (ab256345, Abcam, England, 1:1000), and Ki67 (9129, CST, USA, 1:400), HPR-conjugated secondary antibody (AS014, ABclonal, China, 1:500).

Single-cell transcriptome sequencing analysis

The raw data for the single-cell transcriptomic analysis originated from the research

conducted by Wei Luo[1]. CellRanger (v6.1.2) produced gene expression matrices, transformed into Seurat objects (v4.0.4). DoubletFinder (v2.0.3) eliminated predicted doublets. Cells exceeding gene expression and mitochondrial DNA thresholds were filtered. UMI counts normalized using SCTransform() in Seurat. PCA conducted via RunPCA on SCTransform's variable features. Batch effects in samples were corrected with harmony (v0.1.0). Clusters were identified through nearest-neighbor clustering and dimensionality reduction was done using RunTSNE(). Signature genes in clusters detected with Findmarkers(); cell types annotated based on biomarker and signature gene expression. Tumor cells were re-validated using infercnv (v1.12.0). Finally, cells expressing KIT were annotated.

Western blotting

The cells were lysed by RIPA lysis buffer (P0013B, Beyotime, China) mixed with protease cocktail inhibitor (20109ES05, Yeasen, China). Protein concentration was standardized by BCA Protein Assay KIT (P0009, Beyotime, China). 15μg protein was added to 10% SDS-PAGE gel and then transferred to PVDF membranes. After blocking with 5% skimmed milk for 2 hours, incubation of primary antibodies was carried out at 4°C overnight. Then the membranes were incubated with HRP-conjugated secondary antibody at room temperature for 1 hour. At last, the membranes were detected by ChemiDoc Imaging System (Bio-Rad, USA).

Antibodies involved were as follows: KIT (3074, CST, USA, 1:1000), p-KIT (3391, CST, USA, 1:1000), Akt (9272, CST, USA, 1:1000), p-Akt (9271, CST, USA, 1:1000), MAPK (9102, CST, USA, 1:1000), p-MAPK (9101, CST, USA, 1:1000), GAPDH (A19056, ABclonal, China, 1:1000), HRP-conjugated secondary antibody (AS014, ABclonal, China, 1:5000).

Immunofluorescence (IF)

GIST-T1, GIST-430, GIST-430/654, and GIST-48B cells were cultured on chamber slides to an appropriate confluence. Following this, cells were fixed using 4% paraformaldehyde for 15 minutes and permeabilized with 0.2% Triton X-100 (P0096, Beyotime, China) for 10 minutes. For antigen blocking, a 10% solution of bull serum albumin (BSA) was employed for 60 minutes. This was followed by the application of primary antibodies, which were incubated at 4°C through the night. Thereafter, cells were treated with Alexa Fluor 488-linked secondary antibodies for 60 minutes at room temperature in the dark. Before images were captured using a microscope, slides were stained with DAPI for 10 minutes and washed with PBS for 15 minutes.

Antibodies involved were as follows: c-KIT (3074, CST, USA, 1:1000), Alexa Fluor 488-conjugated secondary antibody (4412, CST, USA, 1:500)

Flow cytometry analysis

The expression level of KIT on the cell membrane surface and the affinity of aptamers were detected using flow cytometry.

To monitor the expression level of KIT, KIT positive cell lines (GIST-T1, GIST-430 and GIST-430/654) and KIT negative cell lines (GIST-48B, HEK293T, BXPC-3, MIAPaPa-2, PANC-1 and PATU8988T) were incubated with PE-labeled IGG (12-4714-82, eBioscience, USA) or PE-labeled anti-KIT antibody (567132, BD Pharmingen, USA) for 1 h at 4°C. After incubation, cells were washed with 1000 μL washing buffer (DPBS supplemented with 4.5 g/L

glucose and 5mM $MgCl_2$) and resuspended in 200 μL binding buffer (washing buffer supplemented with 1 mg/ml BSA and 1 mM yeast tRNA) for flow cytometry analysis.

To monitor the binding affinity, cells mentioned above were incubated with 250nM of Cy5-labeled library, KIT-w, KIT-a, KIT-b, KIT-c, KIT-d, KIT-d-mu1, KIT-d-mu2, KIT-d-mu3, or KIT-d-MMAE for 30 min at 4°C. After incubation, cells were washed with 1000 μL washing buffer and resuspended in 200 μL binding buffer for flow cytometry analysis.

To study the dissociation constant (Kd) of KIT-d, different concentrations of KIT-d were incubated with GIST cells or KIT protein (93764ES80, YEASEN, China) (coated on Aldehyde/Sulfate latex beads). The cells were analyzed by flow cytometry and the K_d was determined by the equation $Y = B\ max\ X/\ (K_d + X)$, using Sigma Plot software (Jandel Scientific).

To assess the affinity of KIT-d for murine KIT, we extracted GIST tumors from mice with spontaneous GIST formation and dissociated them into single-cell suspensions. To minimize the impact of non-GIST cells on the experimental results, we first labeled the tumor cells with ANO-1 antibodies (PA5-119679, Invitrogen, USA) conjugated to FITC fluorescent antibodies (F-2765, Invitrogen, USA), identifying ANO-1 positive cells as GIST cells, which were then incubated with KIT-d as mentioned above.

Fluorescence intensity was measured with BD FACSVerse flow cytometry and analyzed by FlowJo_v10.9. Three independent experiments were performed.

Cell apoptosis was measured by flow cytometry assay with Annexin V-FITC Apoptosis Detection Kit (C1062L, Beyotime, China) according to its protocol. GIST-T1, GIST-430/654, and GIST-48B cells were treated with KIT-d, VcMMAE, or KIT-d-MMAE (0, 10, 25, 50, 100

nM) for 72 h. Then, the apoptotic rates were measured by flow cytometry and analyzed by FlowJo_v10.9. Three independent experiments were performed.

Cell cycle was measured with Cell Cycle and Apoptosis Analysis Kit (C1052, Beyotime, China) according to its protocol. GIST-T1, GIST-430/654, and GIST-48B cells were treated with KIT-d, VcMMAE, or KIT-d-MMAE (0, 10, 25, 50, 100 nM) for 36 h. Then, the cell cycle rates were measured by flow cytometry and analyzed by FlowJo_v10.9. Three independent experiments were performed.

Surface plasmon resonance (SPR)

Multi-cycle kinetics was performed on a BIAcore T200 (GE Healthcare/Cytiva) instrument at 25 °C, following the standard protocol[2] . The His-tagged KIT proteins were immobilized on a CM5 chip, and aptamers diluted to different concentrations were loaded. At the end of each cycle, the surface was regenerated with 50 mM of NaOH for 10 minutes at 30 μL/min, which removed the captured DNA. Data was analyzed using the Biacore Evaluation Software (GE Healthcare/Cytiva).

Serum stability

Serum stability was assessed by incubating 250 nM Cy5-labeled aptamer in cell culture medium containing 10% FBS at 37 °C for varying durations. At designated time points, samples were heat-inactivated and analyzed by electrophoresis.

Computational modeling analysis of the binding of the aptamer to the KIT protein

The construction of the aptamer's 3D structure was executed using RNAcomposer, wherein uracil bases were substituted with thymine, and the ribose's hydroxyl group was eliminated, resulting in a deoxynucleotide framework. To relax the aptamer configuration, a refinement process utilizing molecular dynamics simulations was applied. These simulations were conducted using the AMBER PARM99 force field within the Gromacs software environment. An average structure was derived from a 10 ns MD trajectory analysis. For the 3D structure of the Kit protein, data was retrieved from the RCSB Protein Data Bank (PDB ID: 2EC8) accessible at http://www.rcsb.org. The molecular interaction between the aptamer and Kit protein was investigated through Rosetta8, incorporating both blind and precise docking techniques. The preliminary docking strategy positioned the protein and aptamer approximately 10 Å apart, ensuring a constrained orientation of all heavy atoms in both systems. This approach yielded over 1000 initial docking poses. Subsequent precise docking used these initial positions, permitting flexibility in the side chains of the protein's binding pocket residues and the entire aptamer (excluding terminal base pairs). The optimal conformation, characterized by the lowest binding energy, was selected from more than 100 conformations derived from the precise docking phase.

In the simulation of the protein-aptamer systems, we employed AMBER FF99SB for the protein and AMBER PARM99 for the aptamer[3, 4]. The complexes initially underwent a restraint by a harmonic potential expressed as k $(\Delta x)^2$, where k equals 100 kcal/mol-1Å-2. This step was followed by the optimization of water molecules and counter ions through separate steepest descent and conjugate gradient methods, each for 2500 steps. Subsequently, the entire system was optimized without constraints using the first-step method. This was followed by an

annealing simulation with a mild restraint (k = 100 kcal/mol-1Å-2). The complex systems were incrementally heated from 0 to 298 K over 500 ps in an NVT ensemble, where N represents the number of particles, V is the volume, and T is the temperature, with their product remaining constant. Post-heating, a 10 ns MD simulation was executed at a stable temperature of 298 K and 1 atm pressure, maintaining constant pressure through isotropic position scaling with a relaxation time of 2 ps. The final average structure was determined from 3000 snapshots, which were extracted from the trajectory of the final 3 ns, offering a comprehensive view of the system dynamics.

Confocal microscopy imaging

For the aptamer binding study, GIST-T1 cells were plated in a Cell culture dish (801001, NEST) at a density of 5000 cells/plate. 24 h after seeding, cells were firstly incubated with an anti-KIT antibody (567132, BD Pharmingen, USA) for 1 h at 4°C following incubated with Cy5-KIT-d in binding buffer for 30 minutes at 4°C. After washing twice with DPBS, cells were imaged with a Leica TCS SP8 Confocal Microscope (Leica, Germany). Image analysis was conducted using Image J, focusing on the colocalization between KIT-d and anti-KIT antibodies.

For the internalization study, GIST-T1 cells were incubated with 250nM of Cy5-labeled library, KIT-d, and KIT-d-MMAE in binding buffer for 2 h at 37°C with or without anti-KIT antibody (bs-10005R, Bioss, China) pretreatment. After washing twice with DPBS, cells were incubated with Hoechst 33342 (C01029, Beyotime, 1:100) and cell mask (C37608, Invitrogen, USA, 1:1000) for 20 minutes to stain the nuclear and cell membrane. After washing twice with

DPBS, cells were imaged with a Leica TCS SP8 Confocal Microscope (Leica, Germany). Image analysis was conducted using Image J, and the fluorescence intensity of individual cells was subjected to statistical analysis.

For the endocytic pathway study, GIST-T1 cells were either preincubated with Dynasore (40μM, 2 h), sodium orthovanadate (10μM, 2 h), Wortmannin (5μM, 2 h), Genistein (100μM, 2 h), Nystatin (50μg/mL, 2 h) or kept untreated. After incubation, cells were washed with DPBS and incubated with 250nM of KIT-d-MMAE in a binding buffer for 2 h at 37°C. Finally, Hochest33342 and cell mask were used to stain the nuclear and cell membrane as mentioned above. After washing twice with DPBS, cells were imaged with a Leica TCS SP8 Confocal Microscope (Leica, Germany). Image analysis was conducted using Image J, and the fluorescence intensity of individual cells was subjected to statistical analysis.

For colocalization experiments, GIST-T1 or GIST-430/654 cells were incubated with Lysotracker Green DND-26 (40738ES50, YEASEN, China, 75nM) and 250nM of Cy5-labeled KIT-d-MMAE in binding buffer for 2h at 37°C. Image analysis was conducted using Image J, focusing on the colocalization between KIT-d and Lysotracker Green.

Competition and block assay

We designed two schemes to test the competition between KIT-d and anti-KIT antibodies.

First, GIST-T1 and GIST 430/654 cells were either incubated solely with 250nM Cy5-labeled KIT-d or pre-treated with unlabeled anti-KIT antibody (bs-10005R, Bioss, China) for 30 minutes before the addition of KIT-d. After washing twice with washing buffer, Cy5 fluorescence intensity was measured to test the blocking effect.

Secondly, GIST-T1 and GIST 430/654 cells were either incubated solely with unlabeled anti-KIT antibody or pre-treated with 5μM unlabeled KIT-d for 30 minutes before the addition of anti-KIT antibody. After washing twice with washing buffer, APC-labeled secondary antibody (A-10931. Invitrogen, USA) was added and incubated at room temperature for 30 minutes. After washing twice with washing buffer, APC fluorescence intensity was measured to test the blocking effect. Fluorescence intensity was measured with BD FACSVerse flow cytometry and analyzed by FlowJo_v10.9. Three independent experiments were performed.

Synthesis of ApDC

KIT-d-MMAE was synthesized by coupling thiol-labeled KIT-d with maleimide-modified MMAE. Briefly, the thiol-labeled KIT-d was dissolved in DPBS buffer with 1 mM TCEP for 1 h. After the removal of TCEP, maleimide-modified MMAE with 5-fold equivalent in acetonitrile was added. The mixture underwent continuous stirring at ambient temperature for an overnight duration. Subsequently, it was processed through high-performance liquid chromatography (HPLC) for the purification of ApDCs.

Biological imaging of ApDC *in vivo*

Female BALB/c nude mice (4-6 weeks old; weight 16-20 g) were obtained from Gem Pharmatech (Shanghai, China). GIST-T1 cells ($1*10^6$) were formulated as a 1:1 mixture with Matrigel (BD Biosciences) and were subcutaneously injected into BALB/c nude mice. For *in vivo* imaging, the mice were intravenously injected with Cy5-labeled library, KIT-d, and KIT-d-MMAE (1.0 nmol in 100μL of DPBS) (n = 3 per group). The fluorescence images were

captured by IVIS® Lumina Imaging System III (PerkinElmer, Inc., MA, USA) at 0, 0.5, 1, 2, 3, and 4 h after drug administration. At 4 h, mice were euthanized and fluorescence images of the main organs and tumors were performed. All images were analyzed using the Living Image software v4.4 (Caliper Life Sciences, Inc., Hopkinton, MA, USA).

Male BALB/c nude mice (4-6 weeks old; weight 20-25 g) were obtained from Gem Pharmatech (Shanghai, China). GIST tissues from patients with multi-TKI resistance were implanted into nude mice. The subsequent experimental procedures were carried out as described above.

For long-term imaging studies, GIST-T1 subcutaneous tumor-bearing nude mice were intravenously injected via the tail vein with Cy5-labeled KIT-d-MMAE (7.5 nmol in 100 μL of DPBS). In vivo fluorescence imaging was performed at designated time points (1 h, 2 h, 4 h, 6 h, 12 h, 24 h, and 48 h) (n = 3 per group) post-injection, followed by ex vivo imaging of major organs.

Cytotoxicity detection

Viabilities of treated cells were measured with CCK8 Cell Counting Kit (A311, Vazyme, China) followed by delineated IC50 (Half maximal inhibitory concentrations) curves and proliferative curves. Absorbance was measured at 450 nm by a microplate reader (Synergy 2, BioteK, USA). For IC50 curves, 10000 cells were seeded in 96-well plates and culture for 24 h. Then, the cells were treated with KIT-d, VcMMAE, Lib-MMAE, KIT-d-MMAE, SMCC-DM1, or KIT-d-DM1 at different drug concentrations for 4 days. For proliferative curves, 3000 cells were seeded in 96-well plates and cultured for 24 h. Then, the cells were treated with KIT-

d, VcMMAE, or KIT-d-MMAE (100 nM), and cell viabilities were recorded every two days. IC50 was calculated by GraphPad 9.

Colony formation assay

To evaluate the survival ability of tumor cells after drug treatment, colony formation was conducted in our study. GIST-T1, GIST-430/654, and GIST-48B cells were treated with KIT-d, VcMMAE, or KIT-d-MMAE of 10 nM for 24 h. After treatment, 500 cells in 2 ml complete medium were added into 6-well plates. 3 weeks later, forming colonies were fixed in 4% paraformaldehyde, stained with 0.2% crystal violet, and counted on representative images. Three independent experiments were performed.

Transwell migration assay

To assess cell migration, transwell assays were performed using 8.0 μm pore PET membrane inserts (353097, BD Falcon, USA) in 24-well plates. GIST-T1, GIST-430/654, and GIST-48B cells were pretreated with KIT-d, VcMMAE, or KIT-d-MMAE (25 nM) for 24 h. After treatment, 30000 cells in 200 μl serum-free medium were added into the upper chamber, which was placed in a 24-well plate containing 600 μl complete medium. After 48 hours, the cells in the lower chamber were fixed in 4% paraformaldehyde, stained with 0.2% crystal violet, and counted on representative images. Three independent experiments were performed.

TUNEL assay

GIST-T1 cells were treated with PBS, VcMMAE (100 nM), or KIT-d-MMAE (100 nM) for

48h. Then TUNEL assay was measured with One Step TUNEL Apoptosis Assay Kit (C1088, Beyotime, China) according to its protocol. Cells were imaged with a Leica TCS SP8 Confocal Microscope (Leica, Germany). Three independent experiments were performed.

Pharmacokinetics evaluation of KIT-d-MMAE

For pharmacokinetics evaluation of KIT-d-MMAE, GIST-T1 subcutaneous tumor-bearing nude mice were intravenously injected via the tail vein with KIT-d-MMAE (7.5 nmol in 100 μL of DPBS). At designated time points after KIT-d-MMAE administration, mice were euthanized and blood and major organs (heart, liver, spleen, lungs, kidneys, and tumor) were collected. Serum was obtained by centrifugation at 3,000 rpm for 10 min at 4 °C. Tissues were rinsed with PBS, weighed, and homogenized. MMAE concentrations in serum and tissue homogenates were measured by liquid chromatography−tandem mass spectrometry (LC-MS/MS) and normalized to sample weight or volume using a standard calibration curve.

To evaluate the excretion profile of KIT-d-MMAE, mice were housed individually in metabolic cages following drug administration. Urine and feces were collected at predefined time intervals post-injection. Samples were weighed (for feces) or measured by volume (for urine), and stored at −80 °C until analysis. MMAE concentrations in urine and fecal samples were quantified using liquid chromatography–tandem mass spectrometry (LC-MS/MS). The cumulative amount of MMAE excreted was calculated to determine the excretion pattern of KIT-d-MMAE.

Biosafety assessment of ApDC

Female ICR mice (6-8 weeks old; weight 25-30 g) were obtained from Gem Pharmatech (Shanghai, China). The mice were randomized into four groups with five mice per group: DPBS, 1-day, 14-days and 28-day. The 1-day, 14-day, and 28-day groups were administered KIT-d-MMAE (4 mg/kg) via tail vein injection 1, 14, and 28 days before euthanasia, respectively. The DPBS group was administered with DPBS. All mice were killed at the same time. Blood samples were taken from the mice for biochemical and hematological analyses and the major organs of the mice were collected and subjected to histological examinations.

Female BALB/c nude mice (4-6 weeks old; weight 16-20 g) were obtained from Gem Pharmatech (Shanghai, China). GIST-T1 cells ($1*10^6$) were formulated as a 1:1 mixture with Matrigel (BD Biosciences) and were subcutaneously injected into BALB/c nude mice. The mice were randomized into two groups: DPBS and KIT-d-MMAE. Mice in the KIT-d-MMAE group received 4 mg/kg of KIT-d-MMAE via tail vein injection every four days for a total of six doses (q4d × 6). All mice were sacrificed at the same time point. Blood samples were collected for hematological, biochemical, and cytokine analyses, and major organs were harvested for histological examination.

Antitumor activities of ApDC in GIST cell line xenograft models

Female BALB/c nude mice (4-6 weeks old; weight 16-20 g) were obtained from Gem Pharmatech (Shanghai, China). GIST-T1 cells ($1*10^6$) or GIST430/654 ($3*10^6$) were formulated as a 1:1 mixture with Matrigel (BD Biosciences) and were subcutaneously injected into BALB/c nude mice. The resulting tumors were measured twice a week and the total volume was using the following formula: (length × width^2)/2, where length was the longest

axis and width was the distance perpendicular to the length.

For the GIST-T1 xenograft model, when the tumor volume reached 80–120 mm^3, mice were randomized into six groups (n = 5 per group) and injected via tail vein with DPBS, KIT-d, SMCC-DM1, KIT-d-DM1, VcMMAE or KIT-d-MMAE (equivalent KIT-d-MMAE concentration = 4 mg kg^{-1}) twice a week for 5 times. After treatment, all mice were killed and their tumor and major organs were collected and subjected to immunohistochemical and histological examinations.

For GIST-430/654 xenograft model, when the tumor volume reached 80-120 mm^3, mice were randomized into five groups (n = 5 per group) and administered DPBS, KIT-d, VcMMAE or KIT-d-MMAE (equivalent KIT-d-MMAE concentration = 4 mg/kg) via tail vein injection twice a week for 6 times or imatinib (50 mg/kg) orally once a day for 3 weeks. After treatment, all mice were killed and their tumor and major organs were collected and subjected to immunohistochemical and histological examinations.

For the head-to-head efficacy comparison between the antibody-drug conjugate and the aptamer-drug conjugate, GIST-T1 xenograft-bearing mice were enrolled when tumor volumes reached 35-70 mm^3. Mice were randomized into three groups (n = 6 per group) and treated via tail vein injection with DPBS, DS-6157a (4 mg/kg), or KIT-d-MMAE (4 mg/kg), administered every 4 days for a total of six doses (q4d × 6).

Antitumor activities of ApDC in a GIST PDX model

Male BALB/c nude mice (4-6 weeks old; weight 20-25 g) were obtained from Gem Pharmatech (Shanghai, China). GIST tissues from patients with multi-TKI resistance were

implanted into nude mice. The resulting tumors were measured twice a week. When the tumor volume reached 80-120 mm^3, mice were randomized into five groups (n = 5 per group) and administered DPBS, KIT-d, VcMMAE or KIT-d-MMAE (equivalent KIT-d-MMAE concentration = 4 mg/kg) via tail vein injection twice a week for 6 times or imatinib (50 mg/kg) orally once a day for 17 days. After treatment, all mice were killed and their tumor and major organs were collected and subjected to immunohistochemical and histological examinations.

Antitumor activities of ApDC in a GIST spontaneous tumorigenesis model

Female $Kit^{V558del/+}$ mice[5] (4-6 months old; weight 25-30 g) were randomized into two groups (n = 5 per group) and administered DPBS or KIT-d-MMAE (4 mg/kg) via tail vein injection twice a week for 7 times. After treatment, all mice were killed and their tumor and major organs were collected and subjected to immunohistochemical and histological examinations.

Antitumor activities of ApDC in a GIST liver metastasis model

Female BALB/c nude mice (4-6 weeks old; weight 17-20 g) were obtained from Gem Pharmatech (Shanghai, China). GIST-T1 cells ($2*10^6$) were injected into the spleen to construct the liver metastasis model. Two weeks after tumor inoculation, mice were randomized into two groups (n = 7 per group) and administered DPBS or KIT-d-MMAE (4 mg/kg) via tail vein injection twice a week for 6 times. After treatment, all mice were kept for about 3 weeks and finally killed and their livers and other major organs were collected and subjected to histological examinations.

Statistical analysis

All data are presented as the mean ± SD. GraphPad Prism 9.0 (GraphPad Inc., La Jolla, CA, USA) was used to display differences between two groups (Student's t-test) or among three or more groups (one-way ANOVA analysis followed by Fisher's LSD multiple comparisons). A p-value of < 0.05 was considered statistically significant. ns, not significant; ns = not significant. *$P < 0.05$, **$P < 0.01$, ***$P < 0.001$, ****$P < 0.0001$.

Supporting Table

**KIT-w**	GAGGCATACCAGCTTATTCAAGGGGCCGGGGCAAGGGGGGGTA CCGTGGTAGGACATAGTAAGTGCAATCTGCGAA
**KIT-a**	GAGGCATACCAGCTTATTCAAGGGGCCGGGGCAAGGGGGGGTA CCGTGGTAGGAC
**KIT-b**	GGGGCCGGGGCAAGGGGGGGTACCGTGGTAGGACATAGTAAGT GCAATCTGCGAA
**KIT-c**	GAGGCATACCAGCTTATTCAATACCGTGGTAGGACATAGTAAGTGC AATCTGCGAA
**KIT-d**	ATTCAAGGGGCCGGGGCAAGGGGGGGTACCGTGGTAGGACATA GT
**KIT-d-mu1**	ATTCAAGGGGAAGGGGCAAGGGGGGGTACCGTGGTAGGACATA GT
**KIT-d-mu2**	ATTCAATTGGCCGGGGCAAGGGGGGGTACCGTGGTAGGACATA GT
**KIT-d-mu3**	ATTCAATTGGAAGGGGCAAGGGGGGGTACCGTGGTAGGACATA GT

Supporting Table 1. The synthesized nucleic acid aptamer's name and corresponding sequence.

Supplementary Figure

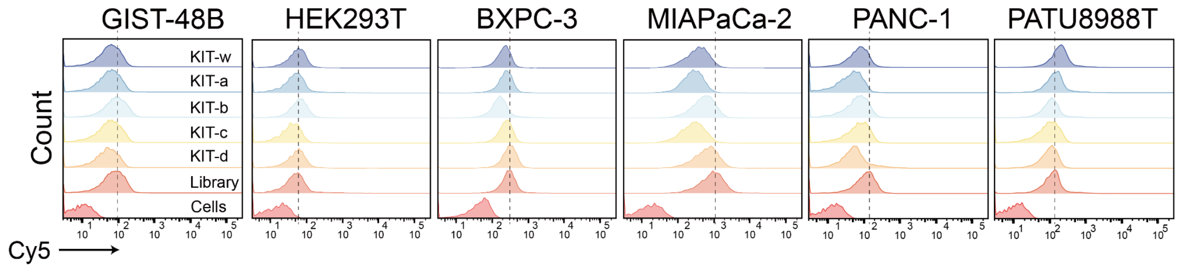

Figure S1. The binding ability of KIT targeting aptamer (Cy5 labeled) to GIST 48B, HEK293T, BXPC-3, MIAPaCa-2, PANC-1, and PATU8988T cells.

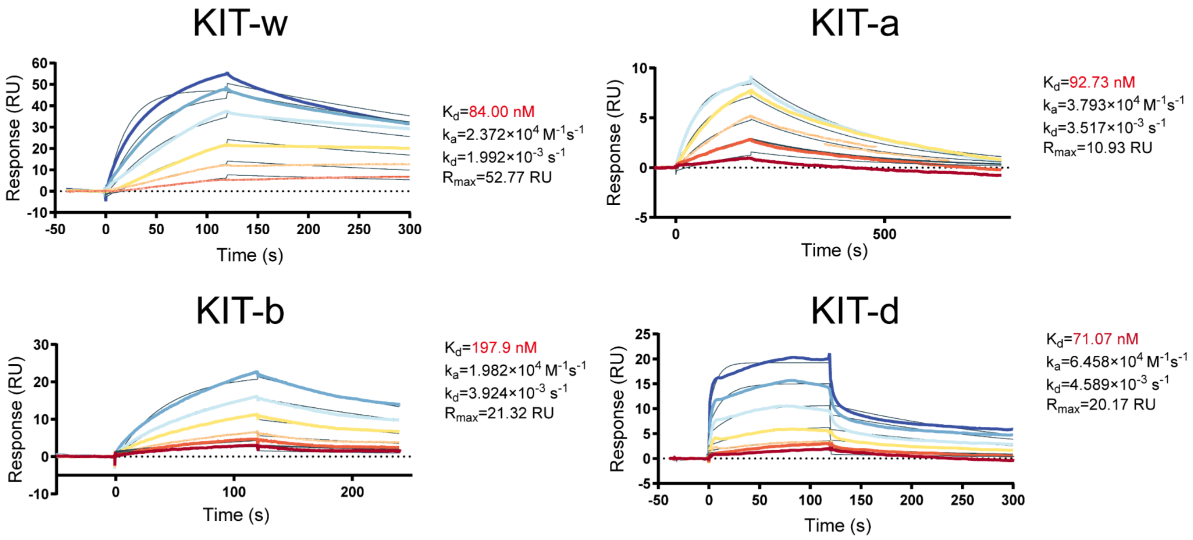

Figure S2. Surface plasmon resonance (SPR) characterizing the binding affinity and kinetics of KIT-w, KIT-a, KIT-b and KIT-d to KIT protein.

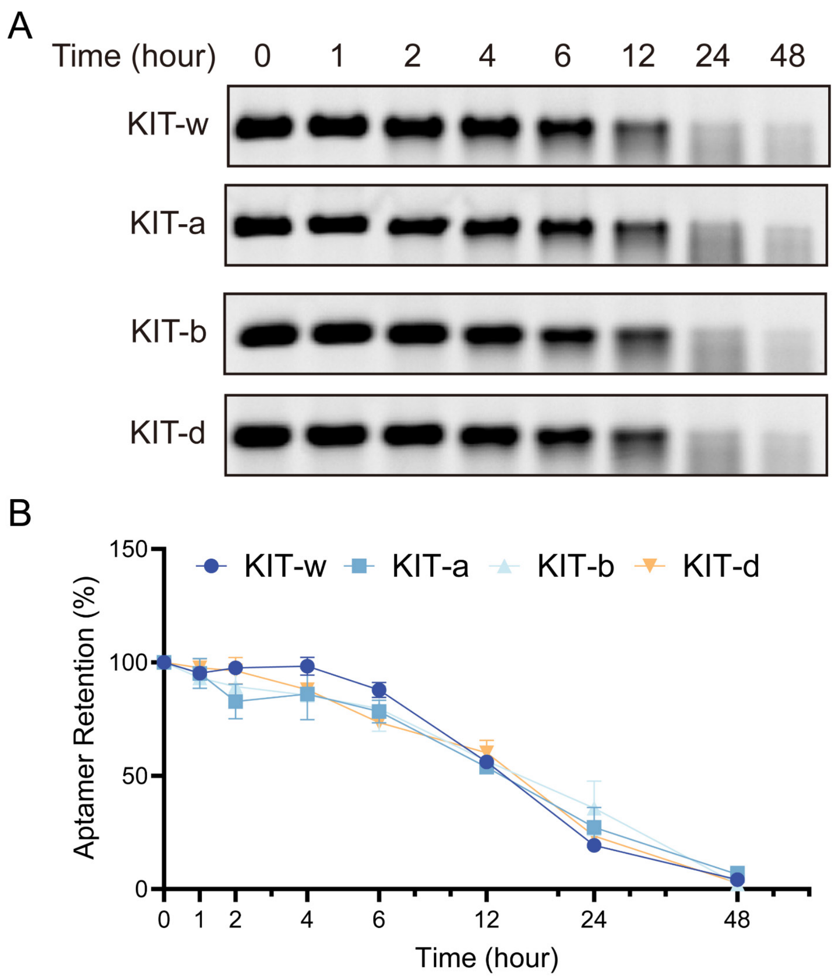

Figure S3. Serum stability of KIT-w, KIT-a, KIT-b and KIT-d. (A) PAGE analysis showing the degradation of each aptamer after incubation in 10% FBS at 37 °C for the indicated times. (B) Band intensity at each time point measured by grayscale analysis from (A).

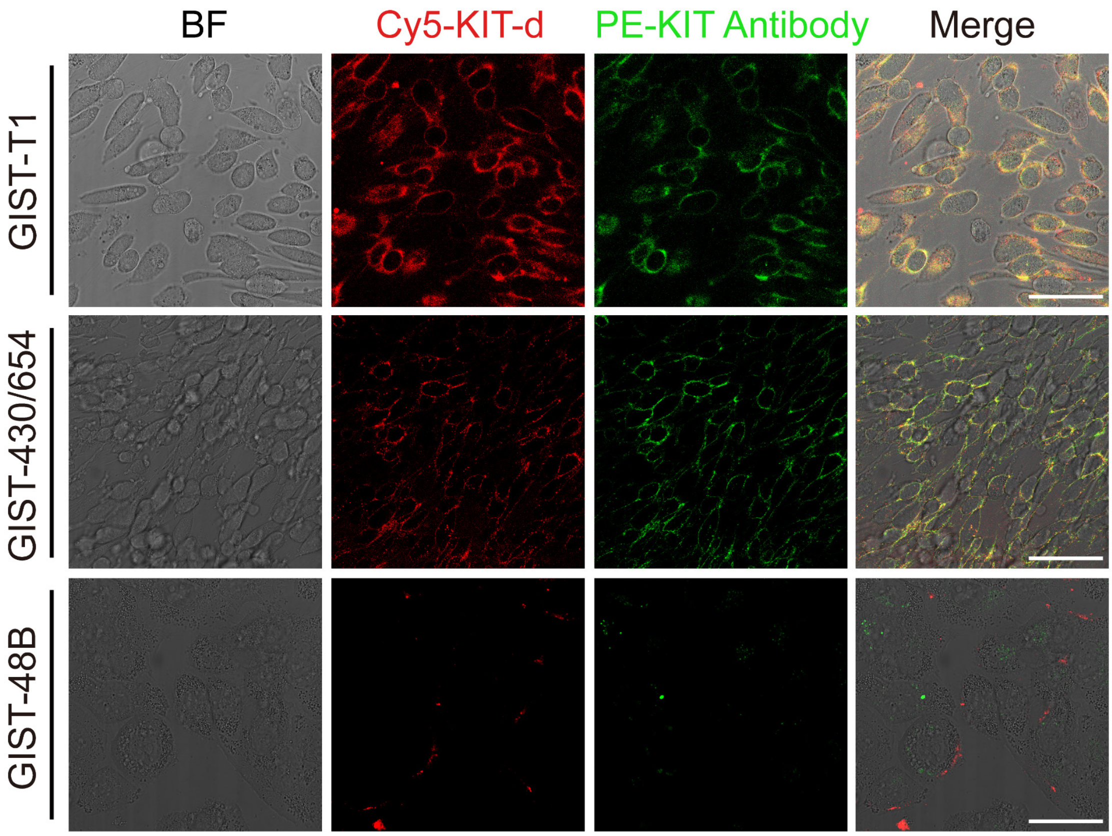

Figure S4. Micrographs showing the binding of KIT-d and KIT antibody to GIST-T1, GIST-430/654, and GIST-48B cells. Cy5-labeled KIT-d (red) and PE-labeled KIT antibody (green) were incubated with cells at 4°C for 30 minutes. The cells were then visualized by confocal microscopy. Scale bar = 50 μm.

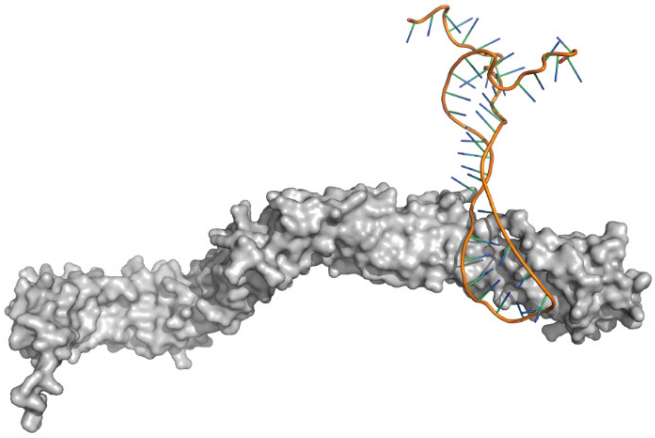

Figure S5. Binding model of the aptamer KIT-d with KIT protein.

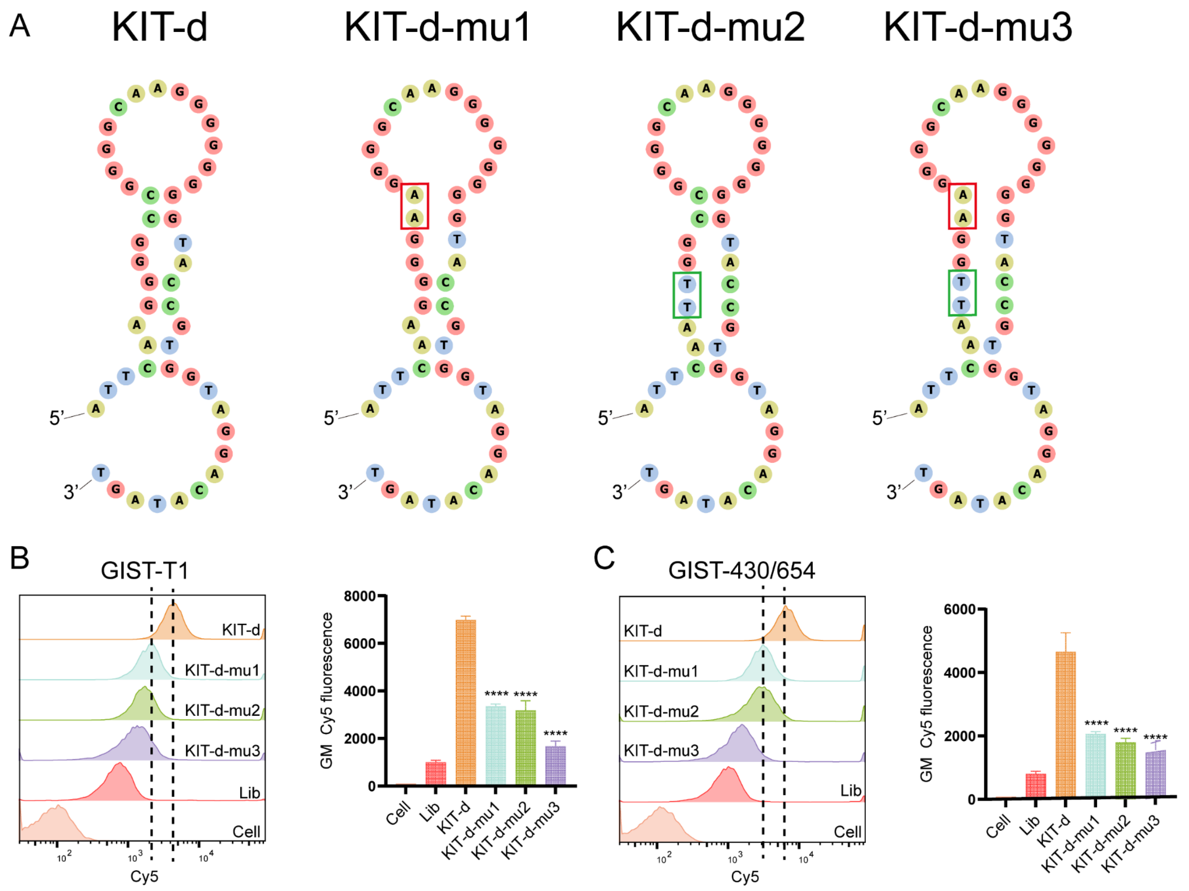

Figure S6. Impact of stem-loop mutations on the binding ability of KIT-d to KIT protein. (A) Predicted secondary structures of KIT-d and its three mutants, with mutation sites indicated. (B) Flow cytometry analysis showing the binding of KIT-d and its mutants to GIST-T1 cells. (C) Flow cytometry analysis showing the binding of KIT-d and its mutants to GIST-430/654 cells.

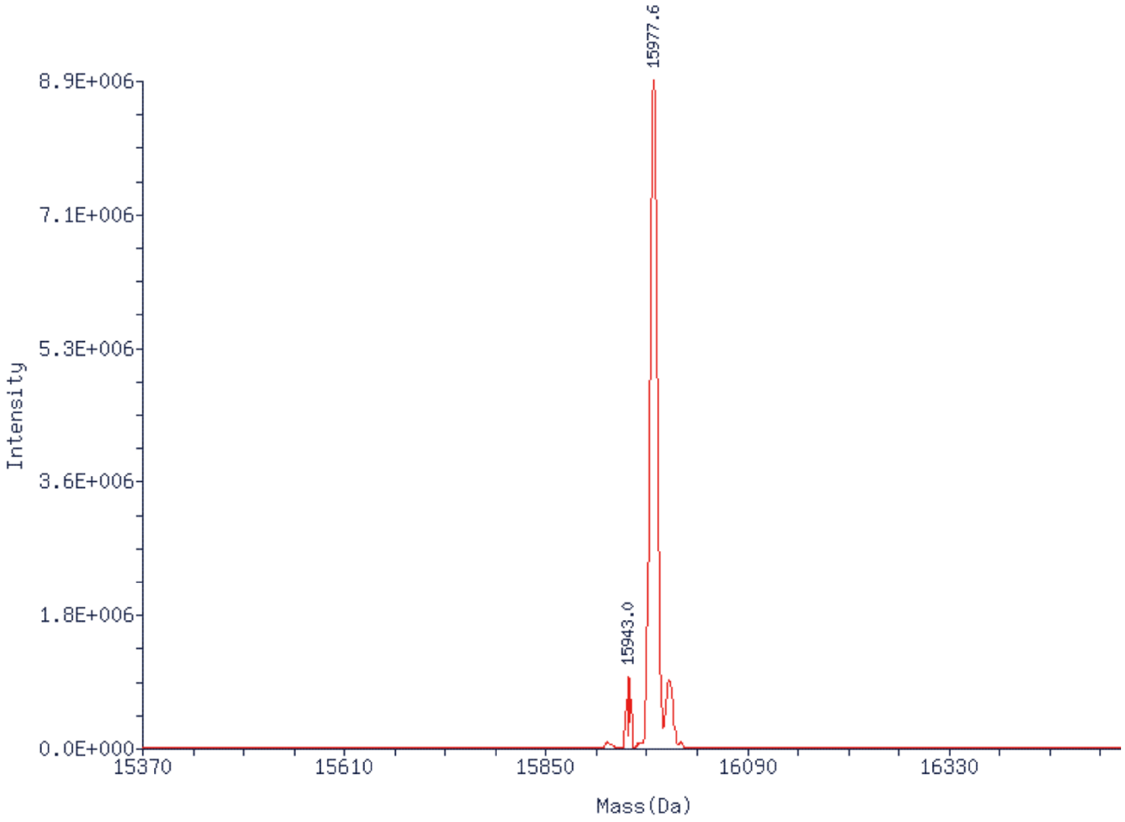

Figure S7. The ESI-MS spectrum KIT-d-MMAE by Biosyntech (Suzhou). The calculated molecular weights were: 15977, and the observed RNA peaks were: 15977.6.

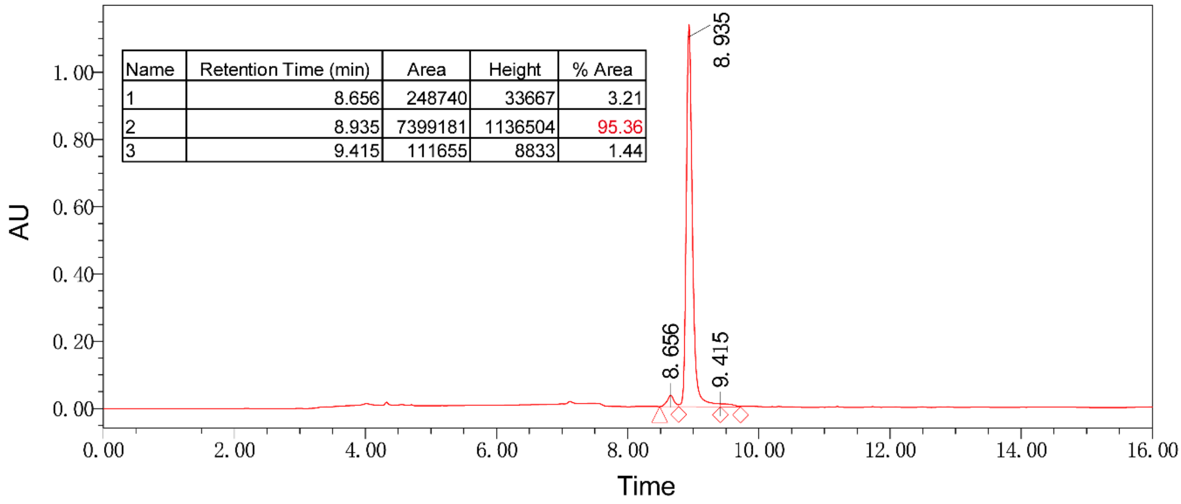

Figure S8. The HPLC chromatogram of KIT-d-MMAE analyzed by Biosyntech (Suzhou). The main peak was observed at 8.935 minutes, indicating the purity of the conjugate.

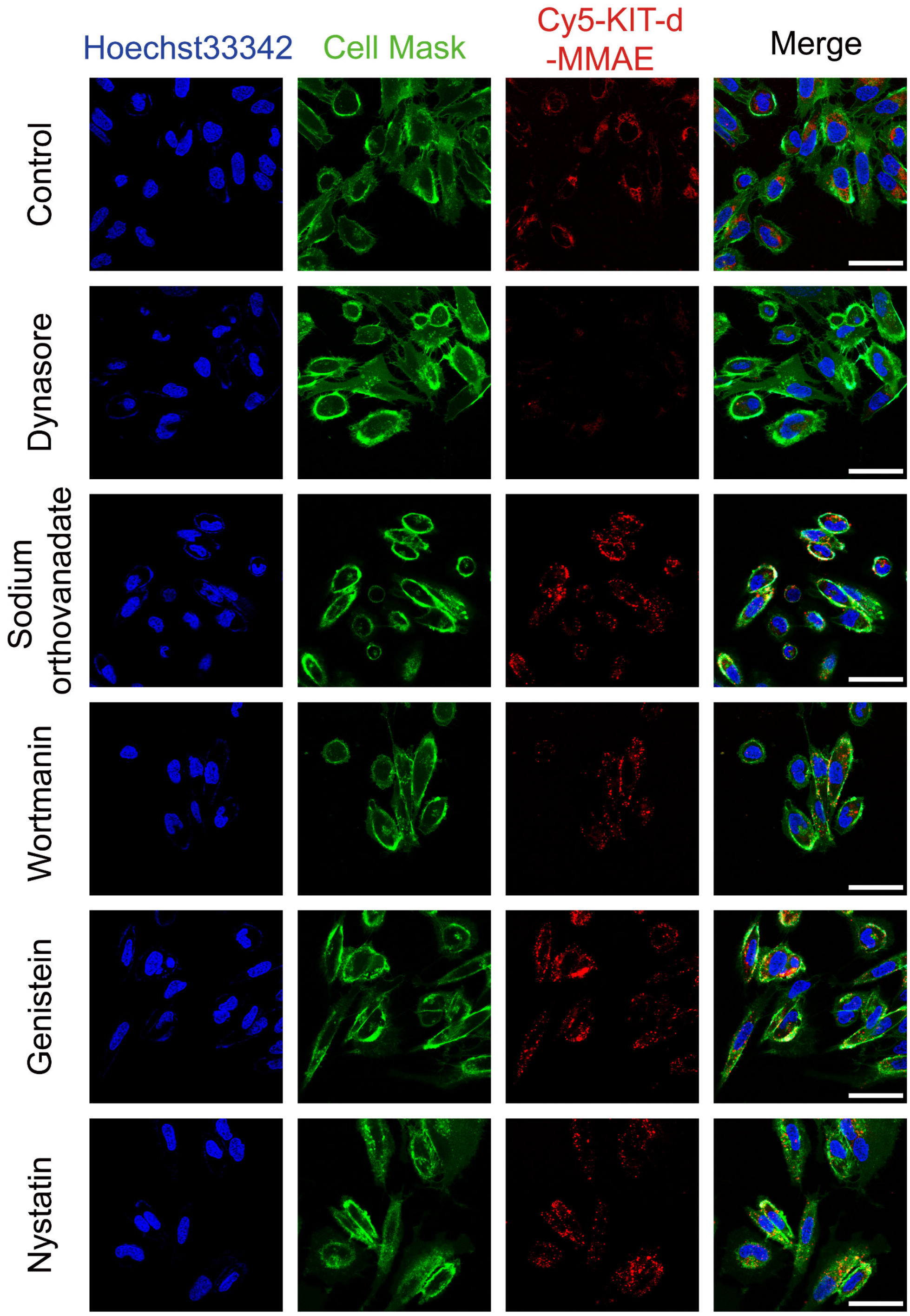

Figure S9. The internalization of KIT-d-MMAE in GIST cells. Confocal microscopy showing the internalization of KIT-d-MMAE-cy5 (red) in GIST cells pretreated with Dynasore (inhibitor of clathrin pathway), sodium orthovanadate (inhibitor of Na^{+}-K^{+} pump), Genistein (inhibitor of the caveolae pathway), Wortmannin (inhibitor of macropinocytosis), Nystatin (inhibitor of lipid raft) or PBS. Hoechst33342 was used to counterstain the nuclei (blue). A cell mask was used to counterstain the membrane (green). Scale bar = 50 μm.

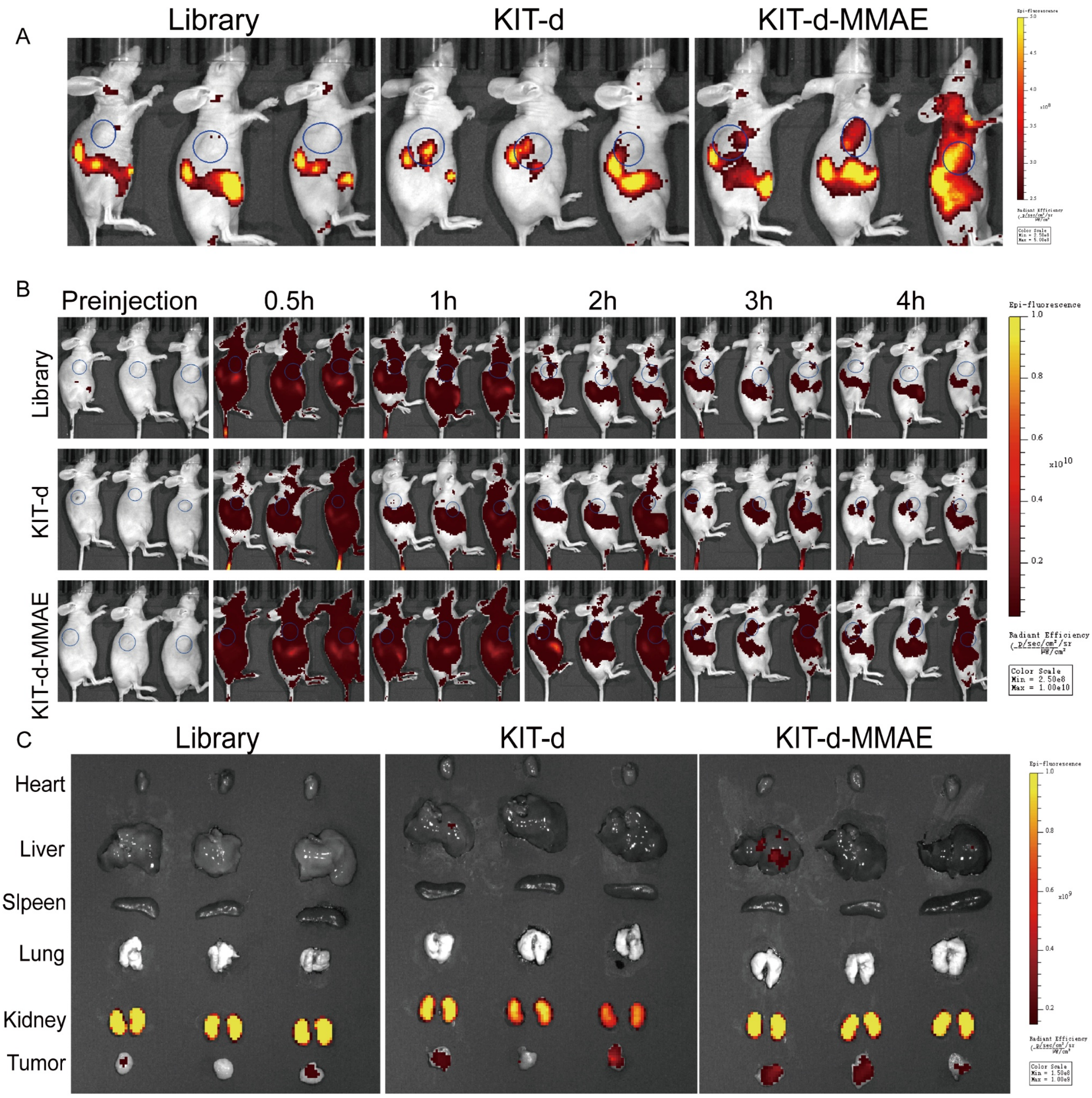

Figure S10. *In vivo* imaging of KIT-d-MMAE. (A) IVIS imaging of GIST-T1 tumor-bearing mice at 4h after intravenously injection with 1 nmol Cy5-labeled library, KIT-d or KIT-d-MMAE (n = 3). (B) IVIS imaging of GIST-T1 tumor-bearing mice at 0, 0.5, 1, 2, 3 and 4 h after intravenously injection with 1 nmol Cy5-labeled library, KIT-d or KIT-d-MMAE (n = 3). (C) Biodistribution of Cy5-Lib, Cy5-KIT-d, or Cy5-KIT-d-MMAE in the major viscera and tumors at 4 h after injection.

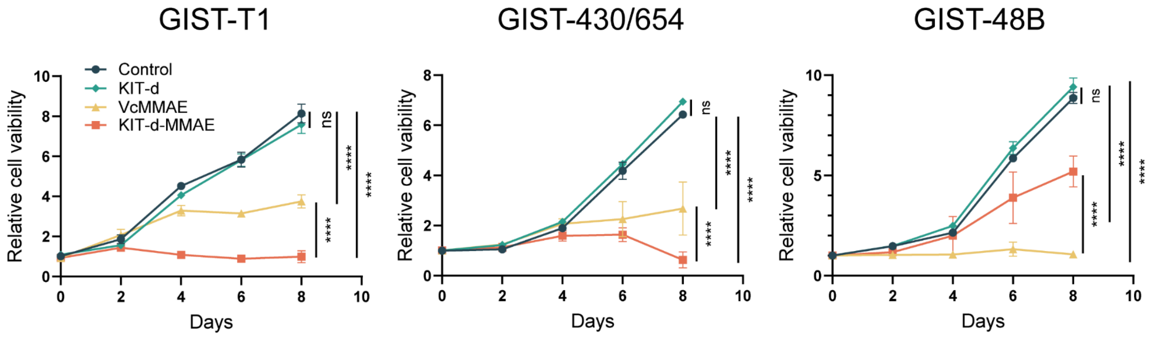

Figure S11. Cell proliferation of GIST-T1, GIST-430/654 and GIST-48B cells with the treatments of drugs (100 nM) was tested with CCK-8 assay. All data are presented as the mean ± SD. ***$P < 0.001$, ****$P < 0.0001$.

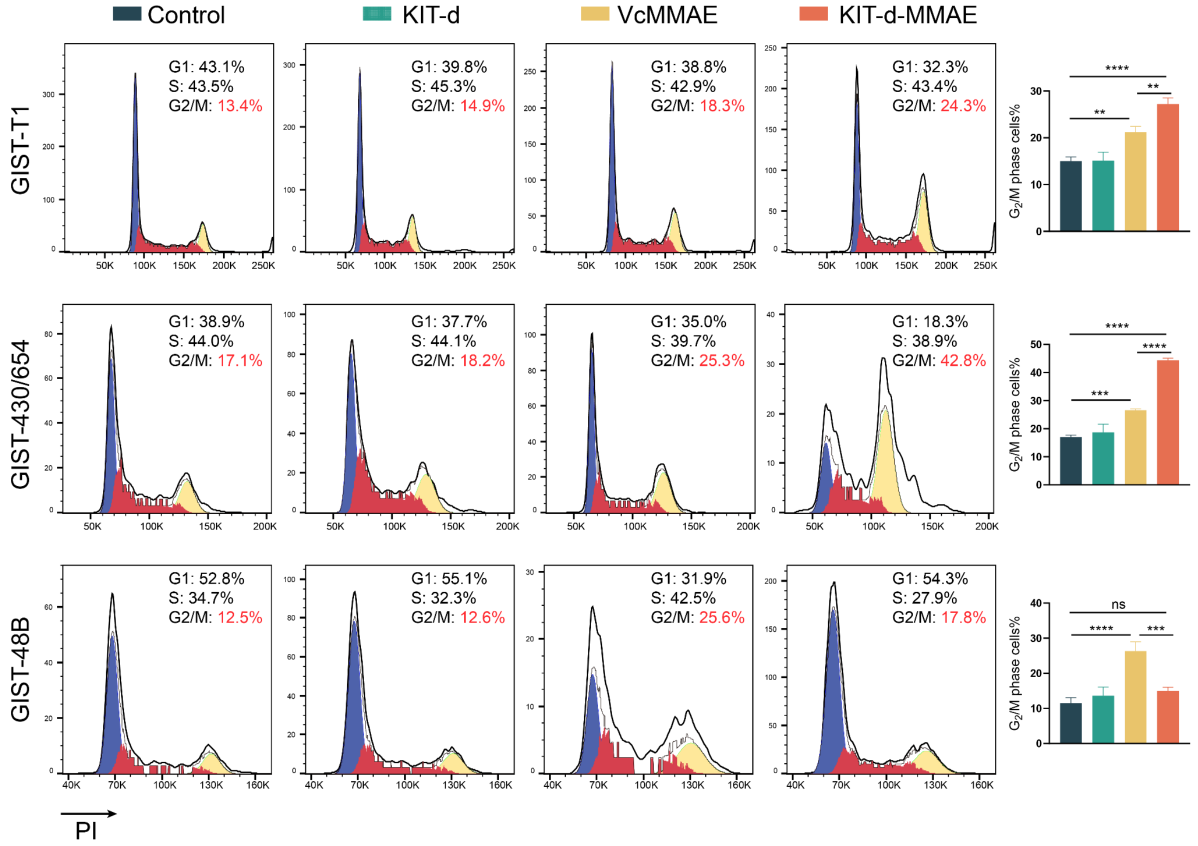

Figure S12. The cell cycle analysis of GIST-T1, GIST-430/654, and GIST-48B cells. The GIST-T1, GIST-430/654, and GIST-48B cells were treated with KIT-d, VcMMAE (100 nM) or KIT-d-MMAE (100 nM) respectively for 48 h with complete medium and then cell cycle was analyzed using flow cytometry. Representative histograms are shown on the left. Quantification of the percentage of cells in G_2/M phase is shown in the chart on the right. All data are presented as the mean ± SD. ns = not significant, **$P < 0.01$, ***$P < 0.001$, ****$P < 0.0001$.

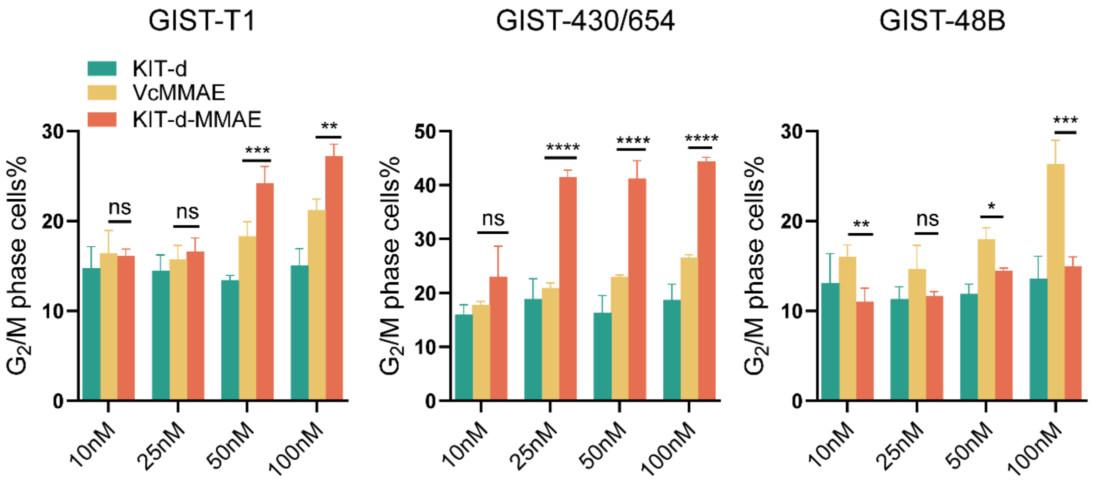

Figure S13. The cell cycle of GIST-T1, GIST-430/654 and GIST-48B cells with the treatment of drugs at different concentrations was analyzed with flow cytometry. All data are presented as the mean ± SD. ns = not significant. *$P < 0.05$, **$P < 0.01$, ***$P < 0.001$, ****$P < 0.0001$.

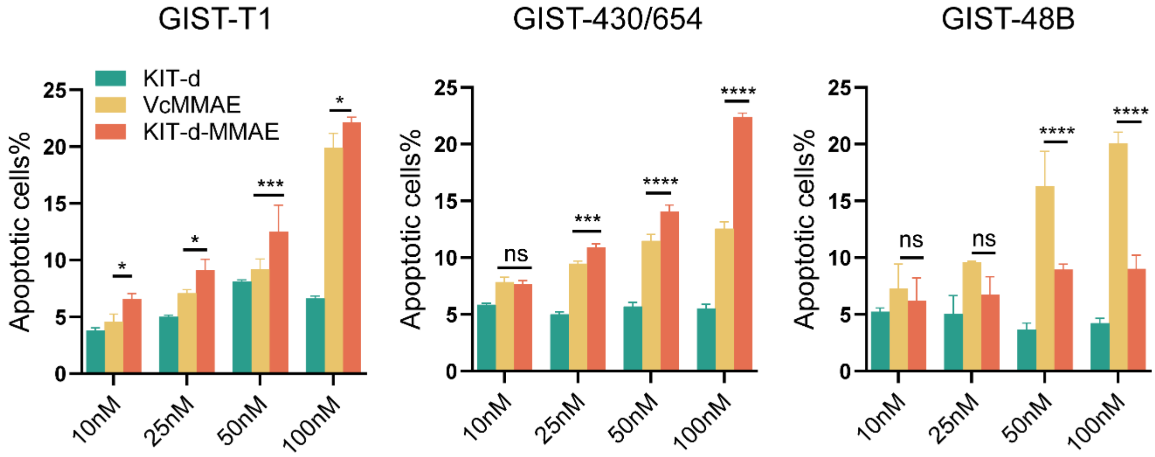

Figure S14. Apoptotic of GIST-T1, GIST-430/654 and GIST-48B cells with the treatments of drugs were studied with flow cytometry, the apoptosis ratio was shown. All data are presented as the mean ± SD. ns = not significant. *$P < 0.05$, **$P < 0.01$, ***$P < 0.001$, ****$P < 0.0001$.

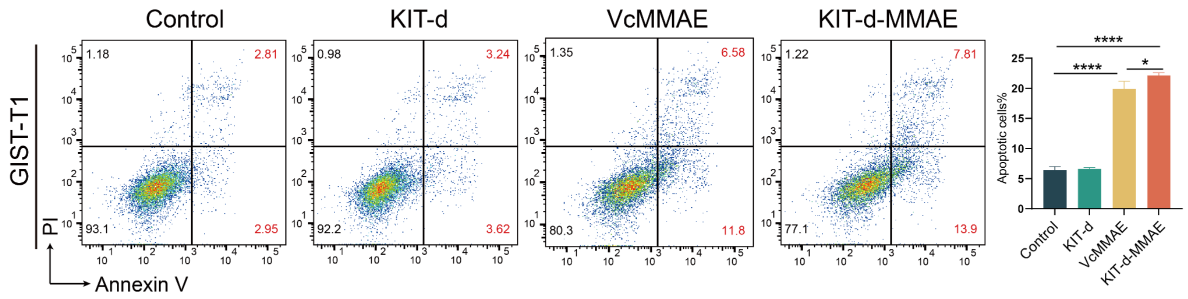

Figure S15. The apoptosis analysis of GIST-T1 cells with the treatment of drugs. The GIST-T1 cells were treated with PBS, KIT-d, VcMMAE, or KIT-d-MMAE (100 nM) respectively for 72 h with complete medium and then analyzed with flow cytometry. The upper right quadrant indicated advanced apoptotic cells and lower right quadrant indicated early apoptotic cells. Corresponding statistical analysis of apoptotic cells were shown in chart on the right. All data are presented as the mean ± SD. *$P < 0.05$, ***$P < 0.001$, ****$P < 0.0001$.

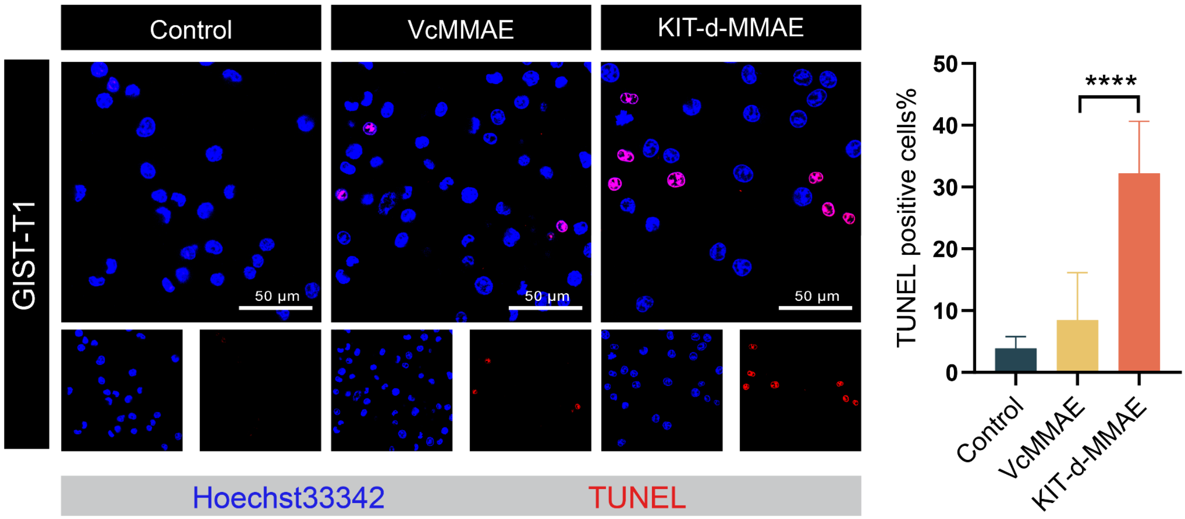

Figure S16. TUNEL assay in GIST-T1 cells. GIST-T1 cells were treated with PBS, VcMMAE or KIT-d-MMAE (100 nM) for 48 h in complete medium, followed by TUNEL staining. Representative fluorescence images are shown on the left. Scale bar = 50 μm. Quantification of TUNEL-positive cells is shown on the right. All data are presented as the mean ± SD. ****$P < 0.0001$.

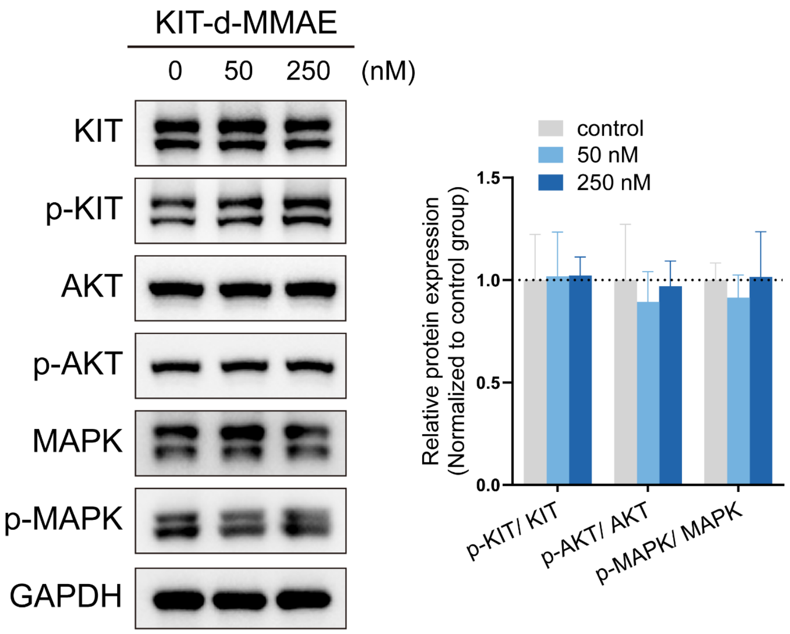

Figure S17. Effect of KIT-d-MMAE on KIT phosphorylation and downstream signaling in GIST-T1 cells. GIST-T1 cells were treated with PBS or KIT-d-MMAE (50 nM or 250 nM) for 48 h. (Left) Western blot analysis of phosphorylated and total KIT, along with key downstream signaling proteins. (Right) Quantification of phosphorylation levels based on densitometric analysis.

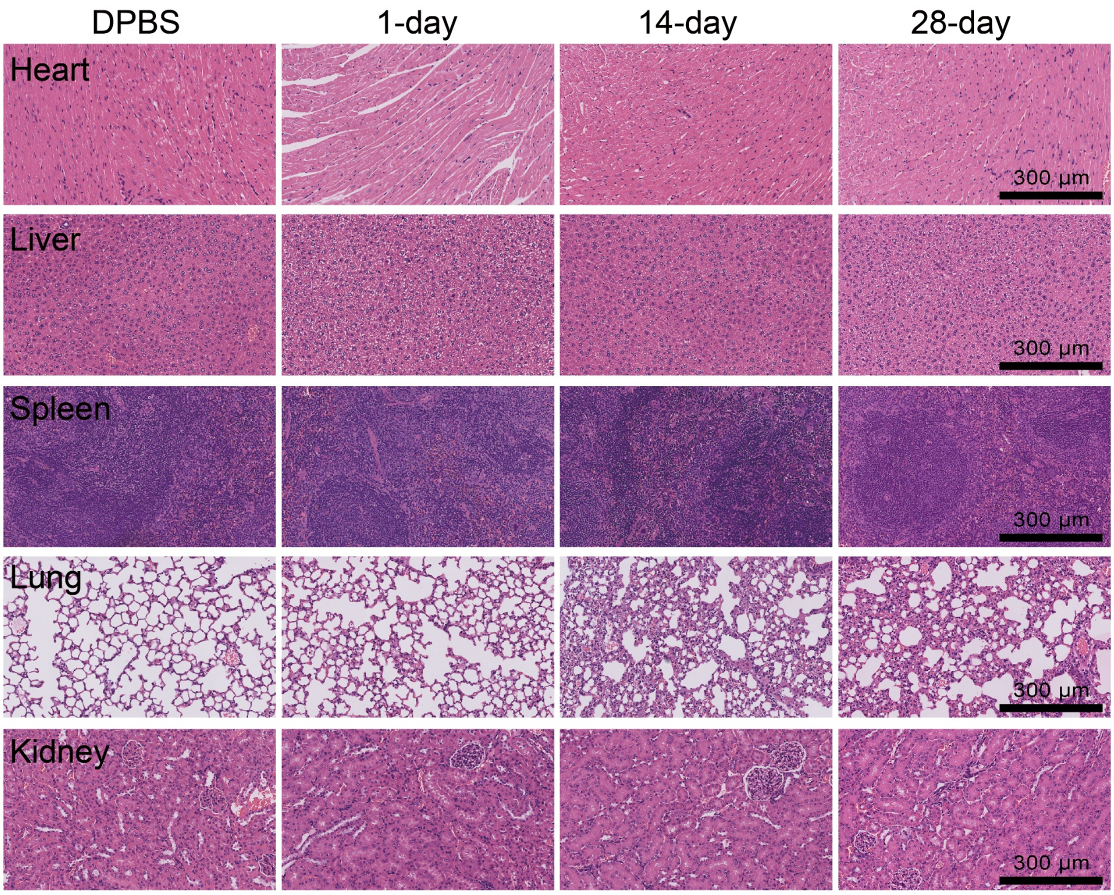

Figure S18. The H&E stain of tissue sections from ICR mice with ApDC treatment for a different time. Scale bars = 300 μm.

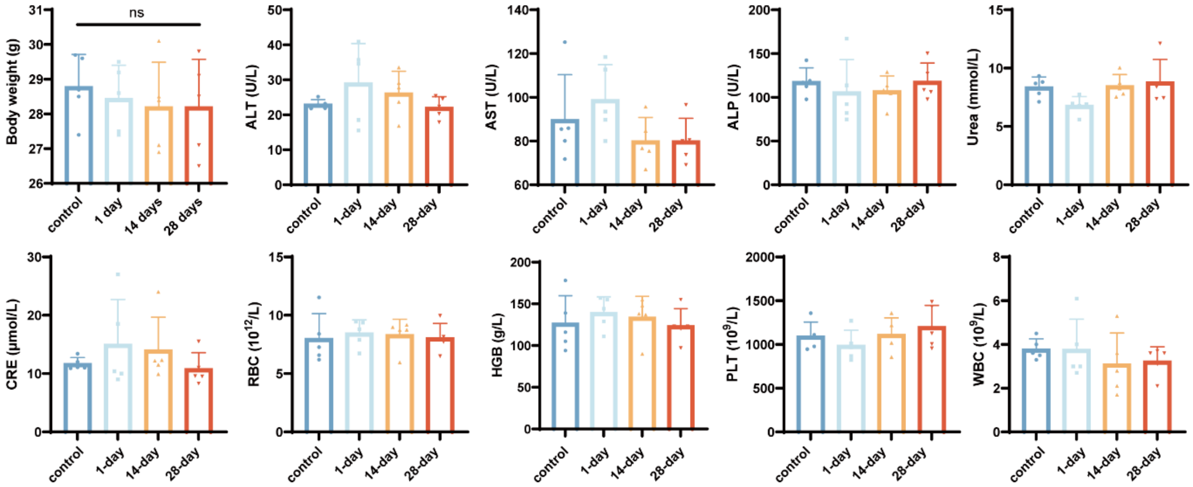

Figure S19. The biosafety assessment of KIT-d-MMAE in ICR mice. Body weight, alanine aminotransferase (ALT), aspartate aminotransferase (AST), alkaline phosphatase (ALP), urea, creatinine (CRE), red blood cells (RBC), hemoglobin (HGB), platelets (PLT), white blood cells (WBC) was analyzed. All data are presented as the mean ± SD. ns = not significant.

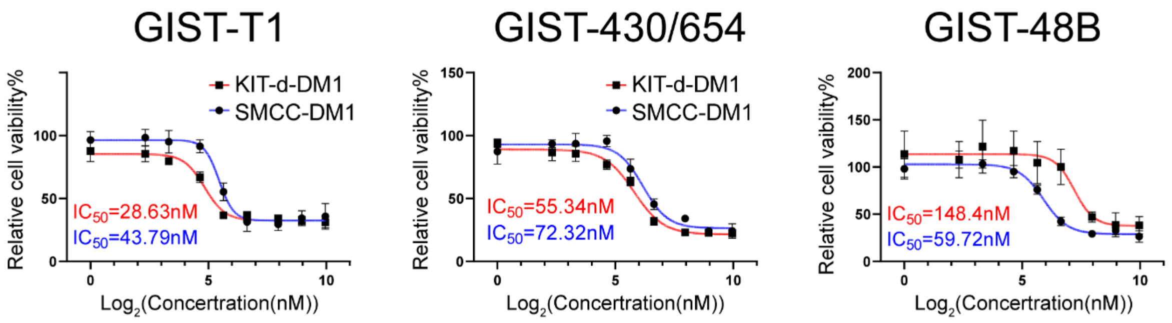

Figure S20. Cell viability of GIST-T1, GIST430/654, and GIST-48B cells with SMCC-DM1, or KIT-d-DM1 treatment. IC_{50} values of SMCC-DM1 (blue) and KIT-d-DM1 (red) are calculated and shown in the chart.

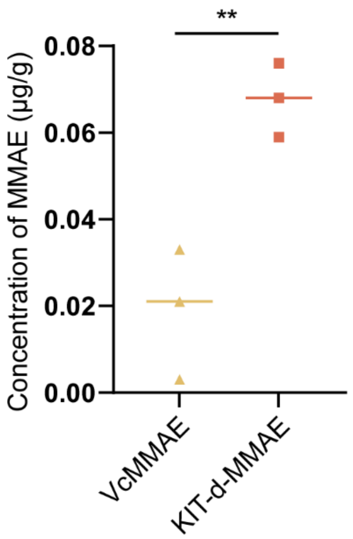

Figure S21. The concentration of MMAE in GIST-T1 xenograft tumor tissues was detected with MS. All data are presented as the mean ± SD. $^{**}P < 0.01$.

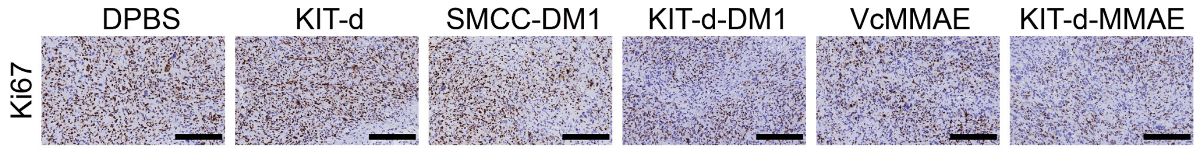

Figure S22. Ki67 staining images of tumor sections from GIST-T1 xenograft tumor model mice with different treatments. Scale bars = 200 μm.

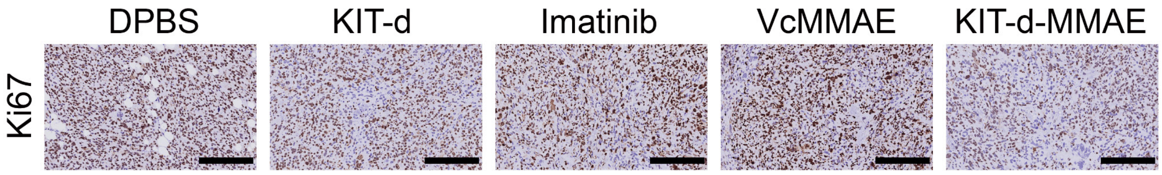

Figure S23. Ki67 staining images of tumor sections from GIST-430/654 xenograft tumor model mice with different treatments. Scale bars = 200 μm

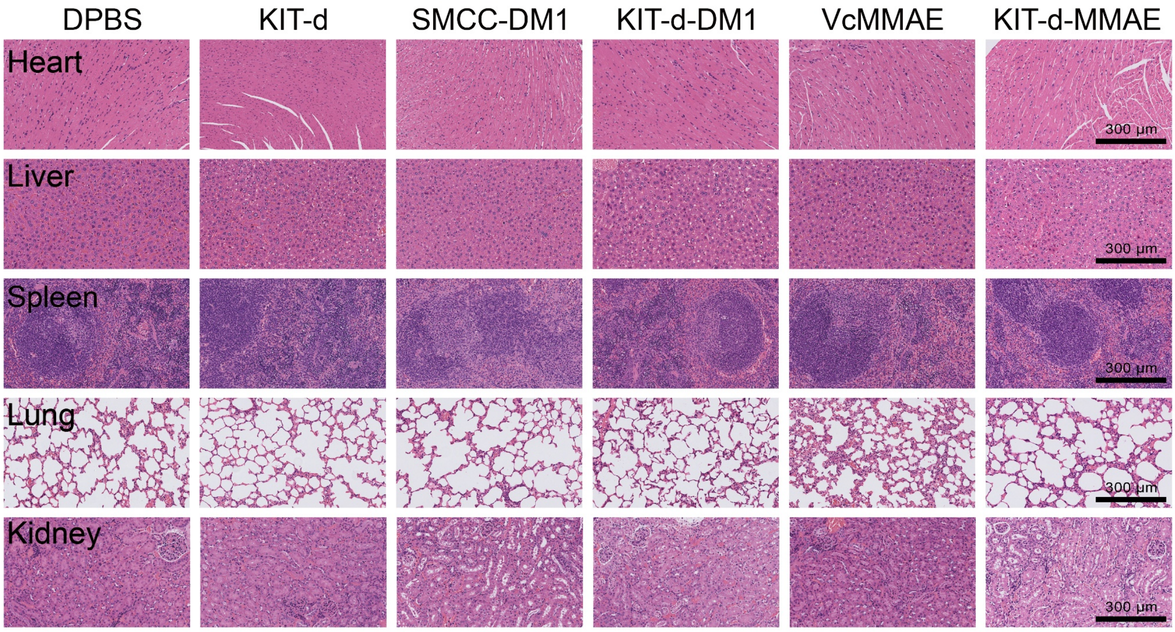

Figure S24. The H&E stain of tissue sections from GIST-T1 xenograft tumor model mice with different treatments. Scale bars = 300 μm.

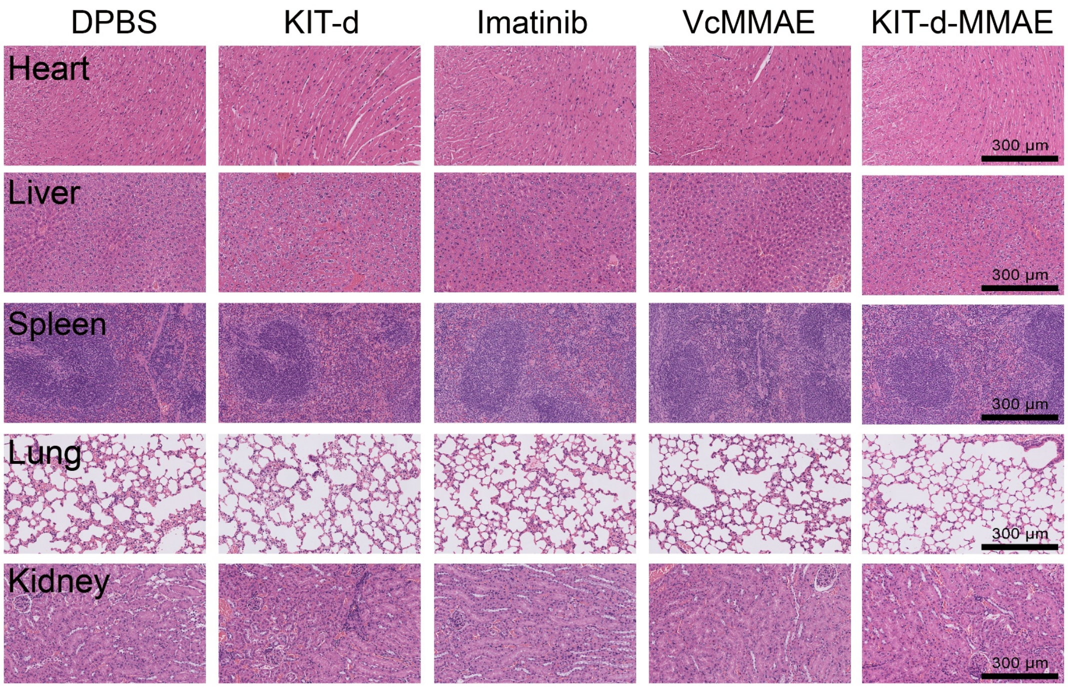

Figure S25. The H&E stain of tissue sections from GIST-430/654 xenograft tumor model mice with different treatments. Scale bars = 300 μm.

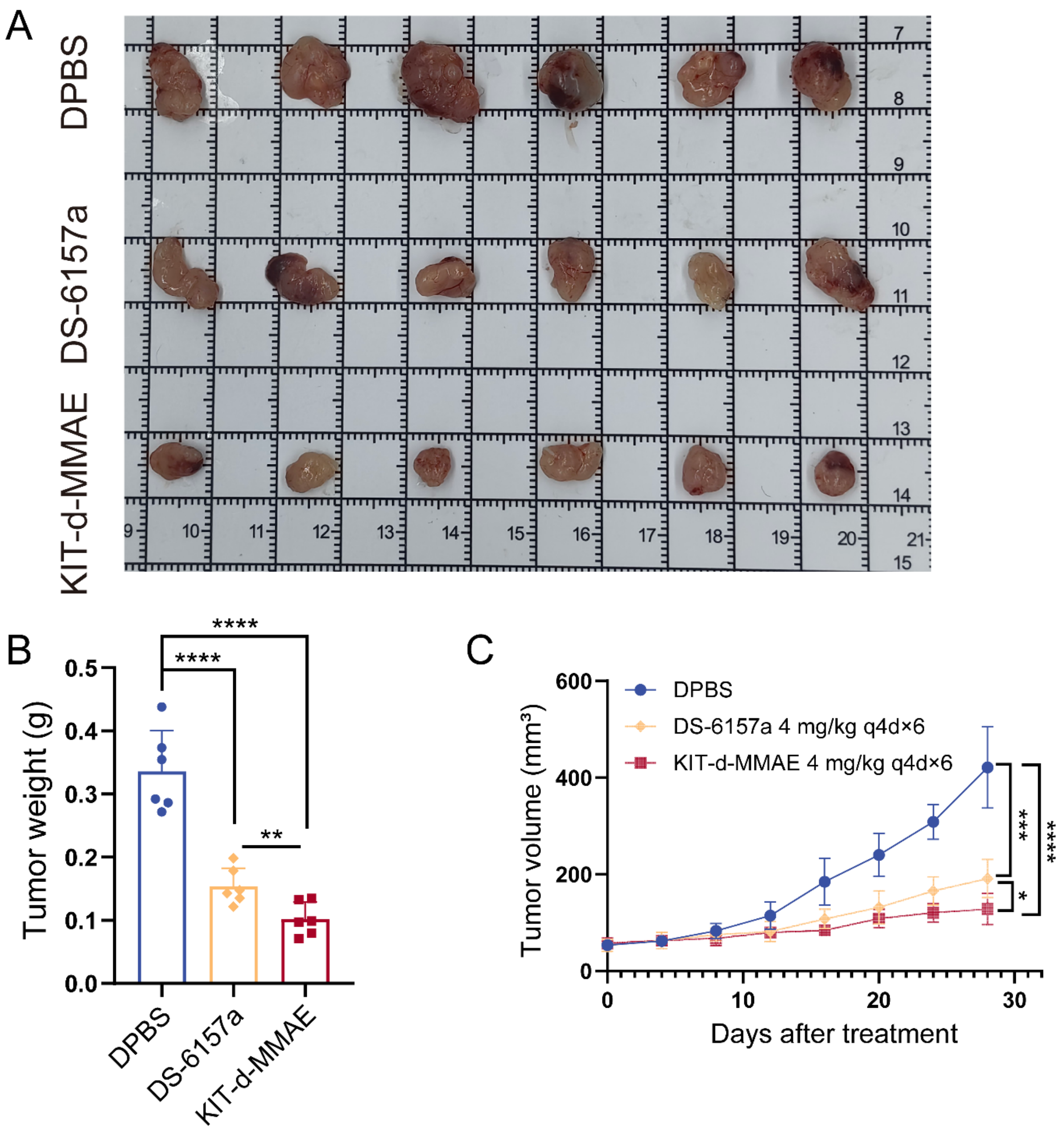

Figure S26. The antitumor efficacy of DS-6157a and KIT-d-MMAE in the GIST-T1 xenograft model (6 mice/group). (A) Images of tumor tissues collected at day 28 post-treatment. (B) Tumor weight of mice after treatment. (C) Growth curve of the tumors during therapy. All data are presented as the mean ± SD. ns = not significant, *$P < 0.05$, **$P < 0.01$, ***$P < 0.001$, ****$P < 0.0001$.

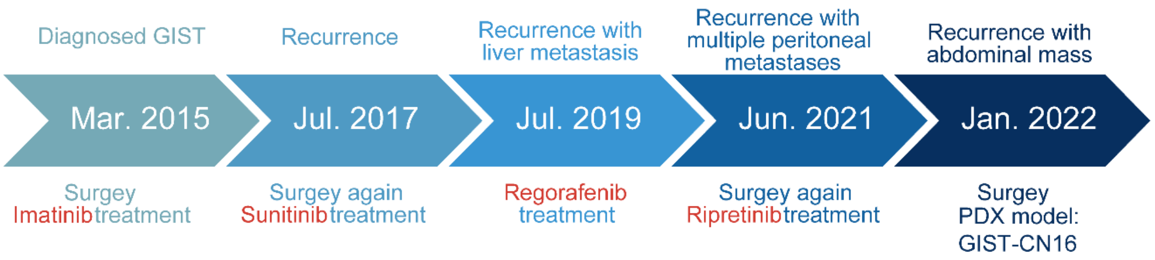

Figure S27. Schematic diagram of the process of patient resistance to multiple TKI drugs.

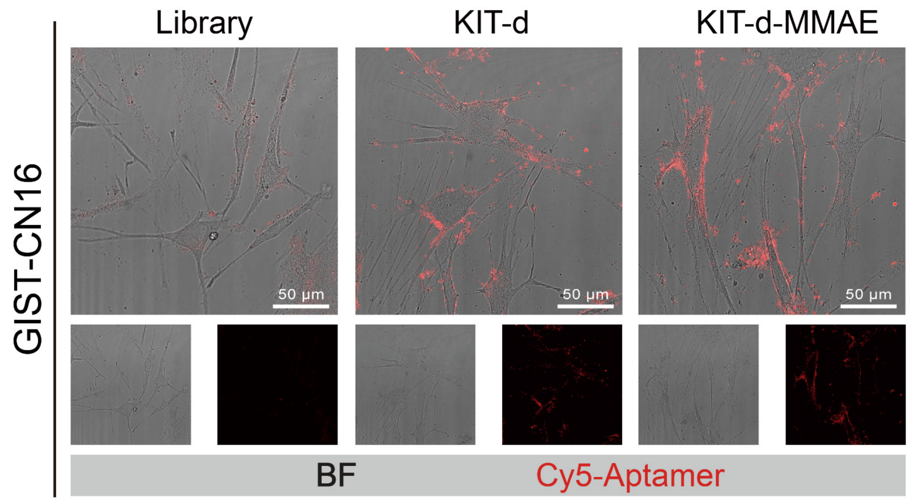

Figure S28. Affinity of KIT-d-MMAE to GIST-CN16. Cells were incubated with Cy5 labeled library, KIT-d or KIT-d-MMAE at 4℃ for 30 minutes. Confocal microscopy showing the binding of Cy5 labeled aptamer to GIST-CN16 cells. Scale bar = 50 μm.

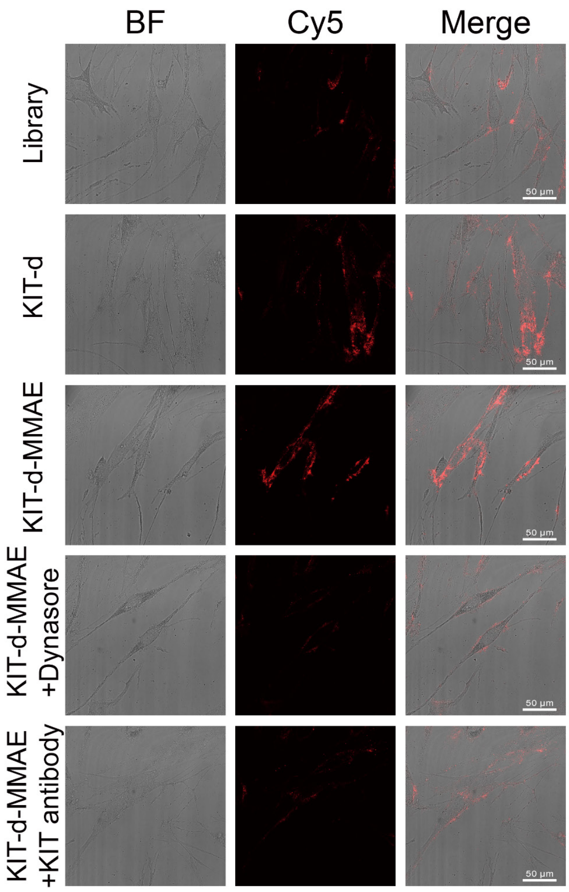

Figure S29. The internalization of KIT-d-MMAE in GIST-CN16 cells. Cells were incubated with Cy5 labeled library, KIT-d or KIT-d-MMAE at 37°C for 120 minutes. Confocal microscopy showing the internalization of library, KIT-d, KIT-d-MMAE, KIT-d-MMAE with dynasore (inhibitor of the clathrin pathway) pretreatment or KIT-d-MMAE with KIT antibody pretreatment in GIST-CN16 cells. Scale bar = 50 μm.

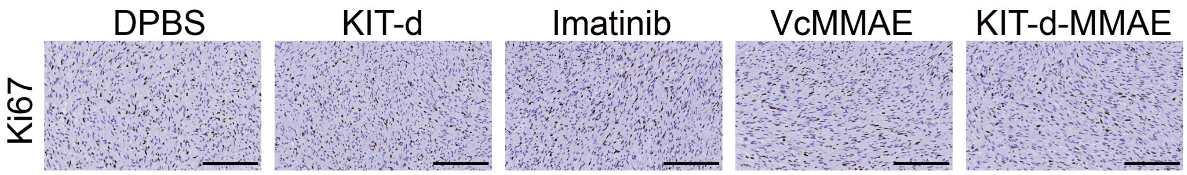

Figure S30. Ki67 staining images of tumor sections from GIST PDX mice with different treatments. Scale bars = 200 μm.

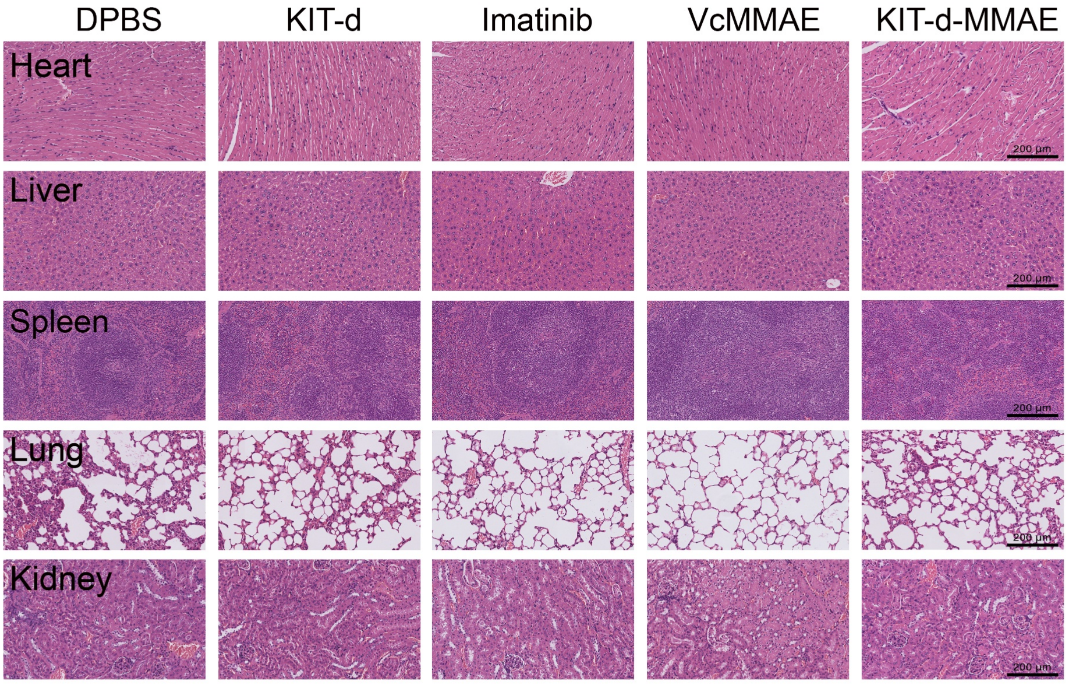

Figure S31. The H&E stain of tissue sections from GIST PDX mice with different treatments. Scale bars = 200 μm.

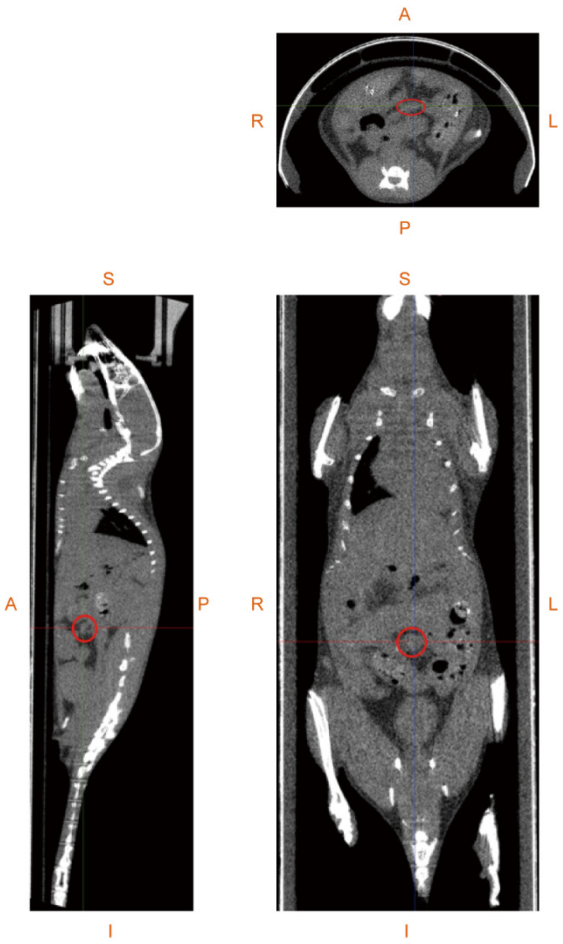

Figure S32. High-resolution CT of mice to verify tumors in GIST spontaneous tumorigenesis model. The tumor (red circle) is located at the ileocecal junction. A: Anterior, P: Posterior, L: Left, R: Right, S: Superior, I: Inferior.

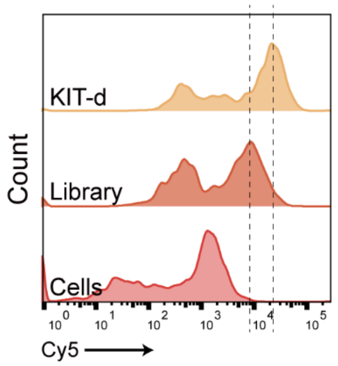

Figure S33. The binding ability of KIT-d (Cy5 labeled) to *Kit*$^{V558del/+}$ cells.

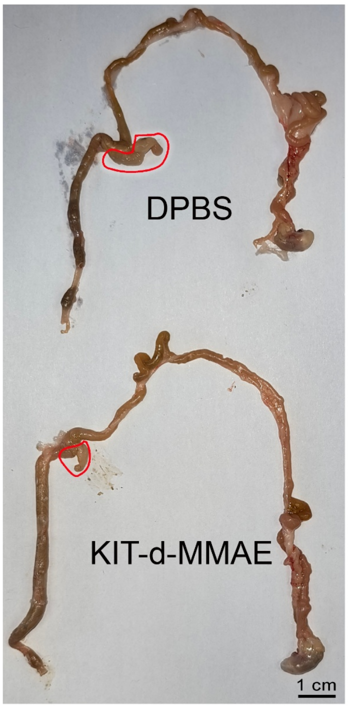

Figure S34. Images of the digestive tract with tumor (red circle) from mice sacrificed at 28 days after the treatment of ApDC.

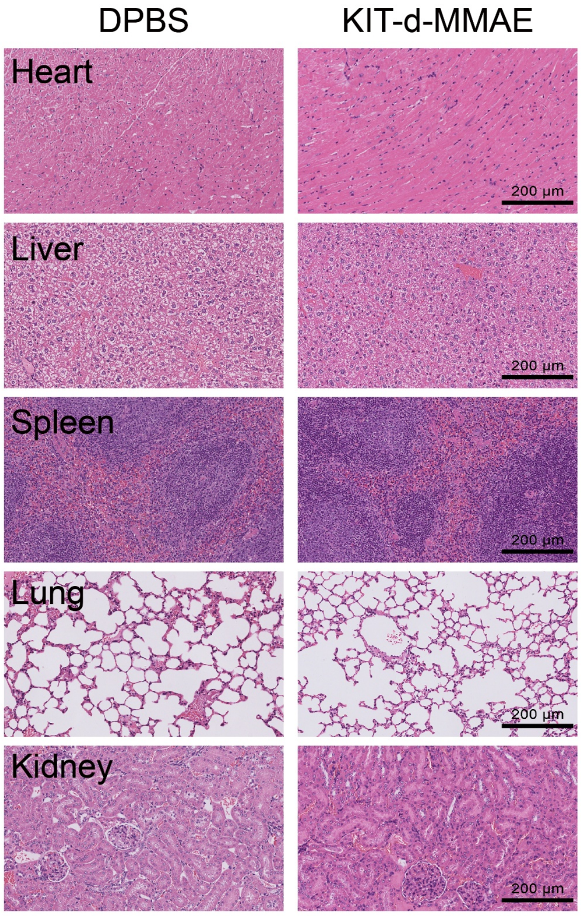

Figure S35. The H&E stain of tissue sections from GIST spontaneous tumorigenesis mice with different treatments. Scale bars = 200 μm.

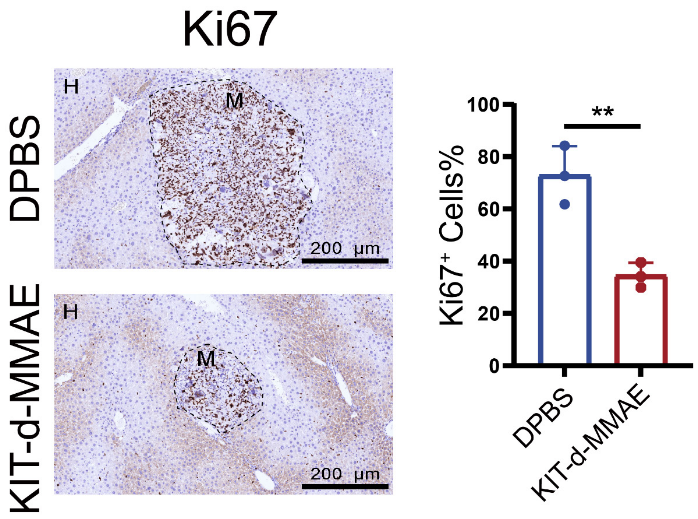

Figure S36. Ki67 staining images of metastatic tumor sections (left) and corresponding statistical charts (right). Scale bars = 200 μm. All data are presented as the mean ± SD. **P <

0.01.

References

1. Mao X, Yang X, Chen X, Yu S, Yu S, Zhang B, et al. Single-cell transcriptome analysis revealed the heterogeneity and microenvironment of gastrointestinal stromal tumors. Cancer Sci. 2021; 112: 1262-74.
2. Chang AL, McKeague M, Smolke CD. Facile characterization of aptamer kinetic and equilibrium binding properties using surface plasmon resonance. Methods Enzymol. 2014; 549: 451-66.
3. Gray JJ, Moughon S, Wang C, Schueler-Furman O, Kuhlman B, Rohl CA, Baker D. Protein-protein docking with simultaneous optimization of rigid-body displacement and side-chain conformations. J Mol Biol. 2003; 331: 281-99.
4. Case DA, Cheatham TE, 3rd, Darden T, Gohlke H, Luo R, Merz KM, Jr., et al. The Amber biomolecular simulation programs. J Comput Chem. 2005; 26: 1668-88.
5. Sommer G, Agosti V, Ehlers I, Rossi F, Corbacioglu S, Farkas J, et al. Gastrointestinal stromal tumors in a mouse model by targeted mutation of the Kit receptor tyrosine kinase. Proc Natl Acad Sci U S A. 2003; 100: 6706-11.
